# Nanoencapsulation of Eucalyptus Essential Oils via Box–Behnken Design: Phytochemical Profiling and Enhanced Antibacterial and Antibiofilm Efficacy

**DOI:** 10.1021/acsomega.5c11998

**Published:** 2026-02-16

**Authors:** Leyla Beba Pozharani, Mehmet İlktaç, Ezgi Ak-Sakallı, Mustafa Alhadi, Ertugrul Ozbil, Azmi Hanoglu, Fatih Demirci, Murat Erdem, Kemal Husnu Can Baser, Muberra Kosar

**Affiliations:** † 52957 Eastern Mediterranean University, Faculty of Pharmacy, via Mersin 10, Famagusta, North Cyprus 99628, Turkiye; ‡ Final International University, Faculty of Pharmacy, via Mersin 10, Kyrenia, North Cyprus 99320, Turkiye; § Near East University, Faculty of Pharmacy, Department of Pharmacognosy, via Mersin 10, Nicosia, North Cyprus 99138, Turkiye; ∥ 52944 Anadolu University, Faculty of Pharmacy, Department of Pharmacognosy, Eskisehir 26470, Turkiye; ⊥ 522675 Eskisehir Technical University, Faculty of Science, Department of Chemistry, Yunusemre Campus, Eskisehir 26470, Turkiye

## Abstract

The increasing burden of infectious diseases and antimicrobial resistance underscores the urgent need for alternatives to conventional therapeutics. Although *Eucalyptus* essential oils (EOs) are well recognized for their broad-spectrum antimicrobial and antibiofilm properties, their relatively high volatility and limited physicochemical stability restrict their practical applications. In this study, a systematic, design-driven comparative approach was employed to develop nanoemulsions containing *Eucalyptus globulus*, *E. citriodora*, and *E. radiata* EOs, with the aim of improving their stability and biological efficacy. Initially, gas chromatography–mass spectrometry was performed to identify species-specific chemotypes, which guided rational formulation design. A Box–Behnken design enabled the precise optimization of critical colloidal parameters, resulting in nanoemulsions with droplet sizes below 200 nm, polydispersity indices below 0.3, and ζ-potentials of approximately −22 mV. Comprehensive structural characterization by Fourier transform infrared spectroscopy and scanning electron microscopy, together with stress testing, confirmed the robust physical stability of the formulations. *In-vitro* antimicrobial evaluations against *Staphylococcus aureus*, *Enterococcus faecalis*, and *Klebsiella pneumoniae* revealed up to a 4-fold enhancement in antimicrobial activity and up to 80% inhibition of biofilm compared to the corresponding unformulated essential oils. The enhancements are most likely attributable to enhanced dispersion, increased interaction with greater microbial interfaces, and nanoscale stabilization of volatile bioactive constituents. Collectively, the optimized *Eucalyptus* nanoemulsions constitute reproducible, chemically well-defined nanocarrier systems with markedly improved antibacterial and antibiofilm performance, highlighting their promise as next-generation delivery systems for phytopharmaceutical applications. Further comprehensive biological and toxicological investigations are required to support the safe and effective translation of essential oil-containing nanocapsulations into practical use.

## Graphical abstract

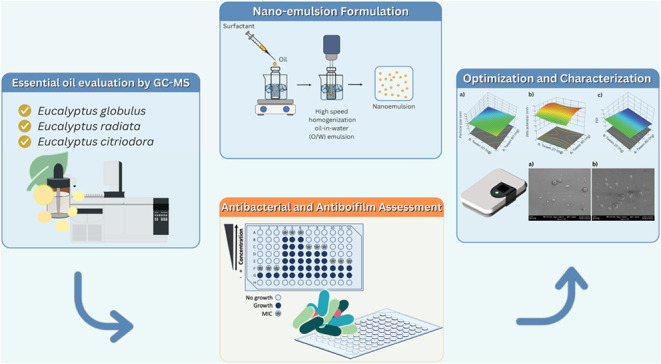

## 1 Introduction

The extensive use of conventional antimicrobials has driven the rapid emergence of resistant pathogens, posing a major global health threat and causing millions of deaths each year. Biofilm-forming bacteria such as *Staphylococcus aureus*, *Staphylococcus epidermidis*, *Enterococcus faecalis*, and *Klebsiella pneumoniae* further complicate treatment by resisting both antimicrobials and host defenses. These growing concerns highlight the urgent need for innovative, sustainable therapeutic alternatives. Among them, plant-derived bioactive compounds offer diverse pharmacological activities and strong potential for integration into advanced drug delivery and biotechnological systems to combat resistant and biofilm-associated infections.^1,2^

Natural compounds from medicinal plants have long attracted interest for their bioactive potential and safety.^3^ Essential oils (EOs), complex volatile mixtures mainly composed of terpenoids and their oxygenated derivatives, exhibit broad-spectrum antibacterial, antiviral, antifungal, and anti-inflammatory properties.^4^ Recognizing their therapeutic promise, the World Health Organization (WHO) encourages further exploration of medicinal plants as alternative tools to address antimicrobial resistance.^5,6^

Phenolic-rich EOs are particularly effective against resistant pathogens, acting through disruption of bacterial membranes, inhibition of efflux pumps and quorum sensing, and suppression of biofilm formation.^7–9^ These mechanisms enhance antibacterial activity while limiting resistance development. Beyond antimicrobial effects, EOs demonstrate anti-inflammatory, antioxidant, and dermal benefits, supporting their utilization in pharmaceutical and cosmetic formulations among other uses.^10^

*Eucalyptus* species (Myrtaceae) and their preparations are widely used as aromatic and medicinal plants, primarily for EOs.^11^ The diverse bioactive profiles of *Eucalyptus* EOs support their therapeutic applications in treating infectious, inflammatory, and respiratory diseases. Among them, *Eucalyptus globulus* Labill. is the major commercial pharmaceutical source, particularly effective against respiratory pathogens.^12^ Other species, including *E. citriodora*, *E. radiata*, *E. smithii*, and *E. dives*, also show remarkable antibacterial and antiviral properties.^13^ EOs rich in 1,8-cineole are commonly used via inhalation for respiratory relief in pharyngitis, bronchitis, and sinusitis due to their anti-inflammatory and broad antimicrobial effects.^14,15^ Notably, *E. globulus*, *E. citriodora*, *E. camaldulensis*, and *E. maculata* demonstrate wide-spectrum antibacterial activity attributed to bioactive constituents such as 1,8-cineole, citronellal, β-cymene, α-pinene, and flavonoids, which disrupt microbial membranes and inhibit pathogen growth.^16^

*E. citriodora* oil, characterized by a high citronellal content (60–80%), is reported to show potent antibacterial, antifungal, antioxidant, and analgesic effects. It is particularly effective against foodborne pathogens such as *Escherichia coli*, *Listeria monocytogenes*, *Salmonella* Enteritidis, and *Staphylococcus aureus*, highlighting its value as a natural aid and preservative agent.^17,18^ Moreover, the major constituents of *E. globulus* and *E. citriodora* 1,8-cineole and citronellal, respectively, have attested *in vitro* inhibitory activity against angiotensin-converting enzyme 2 (ACE2) and lipoxygenase, suggesting potential anti-COVID-19 and anti-inflammatory effects, respectively.^19^

Despite their broad biological activities, EOs face major challenges related to their physicochemical characteristics and physicochemical properties. Their lipophilic nature results in poor aqueous solubility and limited bioavailability in hydrophilic environments. Moreover, their relatively high volatility and tendency of oxidation, isomerization, or polymerization upon exposure to oxygen, heat, or light significantly shorten their shelf life and affect biological as well as pharmacological efficacy. Direct and pure application may also cause irritation or toxicity, restricting therapeutic use. To overcome these limitations, nanoencapsulation offers an effective strategy by enhancing stability, dispersibility, and protection against environmental degradation.^9,20,21^

Beyond improving physicochemical stability, the nanoscale dimensions of EO-based formulations markedly increase surface area, promoting enhanced cellular uptake and closer interaction with microbial membranes, which leads to membrane destabilization, lipid bilayer disruption, and increased permeability, thereby amplifying antimicrobial efficacy. The incorporation of biocompatible excipients, particularly surfactants that are generally recognized as safe (GRAS) by the U.S. Food and Drug Administration (FDA), contributes to high biocompatibility and minimal cytotoxicity, while also supporting environmental sustainability, respectively. Building upon this concept, nanoscale delivery systems provide an advanced means of EO encapsulation, offering superior protection and sustainable platform for developing next-generation antimicrobial substances as well as therapeutics.^22,23^

Encapsulation within sub-200 nm oil-in-water droplets, protects against oxidation and photodegradation, ensures kinetic stability, and enables controlled release. Their small droplet size enhances uptake and antimicrobial action, while steric stabilization by biocompatible surfactants supports safety and sustainability.^9,24^

However, developing a stable nanoemulsion of eucalyptus oil remains challenging due to complex formulation dynamics. Parameters such as oil chemotype, surfactant type and ratio, and emulsification method determine droplet size, stability, and bioactivity.^25^ Although increasing surfactant levels or energy input can minimize droplet size, these changes may degrade thermolabile components, complicating optimization.

Conventional trial-and-error formulation methods are inefficient for understanding the multifactorial interactions that determine stability and performance. In contrast, structured experimental design approaches offer a systematic and efficient means to evaluate multiple variables simultaneously. Among these, the Box–Behnken Design (BBD) is particularly advantageous, as it minimizes experimental runs, models factor–response relationships, and avoids extreme conditions that could compromise sensitive systems.^26–28^ Integrated within the Quality by Design (QbD) framework recommended by the FDA and ICH (International Council for Harmonization of Technical Requirements for Pharmaceuticals for Human Use), BBD enables the establishment of empirical models linking formulation variables to nanoemulsion characteristics, supporting prediction, optimization, and scale-up. For complex systems such as EO nanoemulsions, this statistically driven approach enhances robustness, conserves resources, and facilitates regulatory compliance.^29,30^

The present study aimed to develop and optimize EO based nanoemulsions of three *Eucalyptus* species using the Box–Behnken Design to address the inherent limitations of EOs, such as poor solubility and physicochemical properties. The optimized fomulations were systematically evaluated and characterized for their structural and stability profiles and assessed for their *in vitro* antibacterial and antibiofilm performance against clinically relevant pathogens commonly involved in community- and hospital-acquired, device-related, and wound infections. By coupling nanotechnology with bioactives, this work establishes a new, stable, and scalable nanocarrier platform that potentiates bacterial membrane disruption and suppresses biofilm formation, providing an innovative and sustainable strategy to combat microbial resistance and strengthen future anti-infective development.

## 2 Materials and Methods

### 2.1 Materials

The commercial EOs of *E. globulus*, *E. citriodora*, and *E. radiata* were generously provided by Doallin Ltd., Istanbul (Turkey). Polysorbate 20 (Tween 20), sorbitan monooleate (Span 80), polysorbate 80 (Tween 80), sodium alginate, and dialysis bags (molecular weight cutoff = 60 kDa,) were purchased from Sigma Chemical Co. (St. Louis, MO, USA). Methanol and hexane were acquired from Merck (Darmstadt, Germany), solvents and chemicals used were in high purity. Cellulose acetate syringe-filters with a pore size of 0.45 μm were obtained from ISOLAB (Eshau, Germany). All studies were conducted using double-distilled water.

### 2.2 Determination and Identification of the Essential Oil Constituents

The quality and constituents were verified by Gas Chromatography/Mass Spectrometry (GC/MS) using Agilent 5975 GC-MSD system. HP-Innowax column (60 m × 0.25 mm, 0.25 μm film thickness) was used for the analyses. Carrier gas flow was at 0.8 mL/min. The temperature of the injector was set to 60 °C for 10 min, and then adjusted to reach 220 °C at a rate of 4 °C/min. After the temperature was held at 220 °C for 10 min, it increased to 240 °C at a rate of 1 °C/min and held constant at 240 °C for 20 min. The split ratio was adjusted to 30:1. Injection port was set to 250 °C. Mass spectra (MS) were recorded at 70 eV and obtained in a mass range from *m*/*z* 35 to 450. The results were expressed as relative percentages (%), which were calculated from peak area.

The identification of EO constituents was performed by comparing their retention times with those of authentic reference compounds or by comparing their linear retention indices (LRI) with those of a homologous series of *n-*alkanes (C_8_–C_40_). Additionally, identification was supported through computer-assisted matching using commercial databases such as the Wiley GC/MS Library and the NIST Chemistry WebBook,^31,32^ as well as an in-house database, the “Başer Library of Essential Oil Constituents”, which includes authenticated compounds and components of known EOs. Mass spectral (MS) literature data were also consulted.^33^

### 2.3 Preparation and Optimization of the Nanoemulsion Essential Oil Formulations

The preliminary formulation development studies were initiated by varying the composition of the EOs from different *Eucalyptus* species (*E. globulus*, *E. citriodora*, and *E. radiata* *)* combined with various surfactants (Tween 20, Span 80, and Tween 80) to identify the most effective surfactant and establish the minimum concentration required for forming stable nanoemulsions.

Nanoemulsions of the selected *Eucalyptus* EOs were prepared according to a modified protocol based on previously described methods.^22,34^ Oil-in-water (O/W) nanoemulsions were prepared by sequentially applying low-energy spontaneous emulsification, followed by high-energy homogenization using a homogenizer (Miccra MiniBatch D, Germany) operating at 27,000 rpm. The EOs were individually weighed and added dropwise to the aqueous phase containing sodium alginate and surfactants at varying concentrations, maintained at 4 °C. The mixture was magnetically stirred at 500 rpm for 15 min to obtain a coarse pre-emulsion (Figure 1).

**Figure 1:**
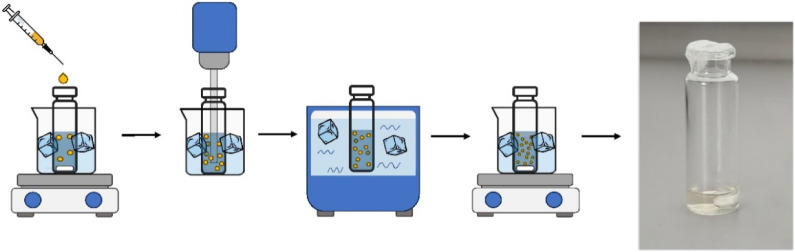
Schematic representation of the EO nanoemulsions preparation procedure.

The final EO concentration in each nanoemulsion formulation was fixed at 7.5% (150 mg per 2000 mg, w/w). The concentrations of Tween 80, Tween 20, and sodium alginic acid were systematically varied at three distinct levels, as detailed in Table 1.

**Table 1:** Formulation Variables and Their Levels Used in the Experimental Design for EO Nanoemulsions

Factor	Name	Units	Type	Minimum	Maximum	Coded Low	Coded High
*X*_1_	Tween 80	mg	50.00	150.00	–1↔ 50.00	+1↔150.00	100.00
*X*_2_	Tween 20	mg	10.00	30.00	–1↔ 10.00	+1↔ 30.00	20.00
*X*_3_	Sodium alginate (q.s. 2000 mg)	%	0.10	1.00	–1↔ 0.10	+1↔ 1.00	0.55

Following the coarse emulsification, the mixture was subjected to high-speed homogenization at 27,000 rpm for 10 min to initiate significant droplet size reduction. Further breakdown of droplets was achieved through ultrasonication at approximately 40,000 Hz for 30 min, utilizing an ultrasonic water bath (Ultrasons H, J.P. Selecta, Spain). Finally, the resulting nanoemulsion formulation was stirred overnight at approximately 500 rpm to ensure uniform dispersion and enhance stabilization.^35–37^

#### 2.3.1 Formulation Optimization of the Essential Oil Nanoemulsions

The optimization of EO nanoemulsion formulations was conducted using Response Surface Methodology (RSM) within a Design of Experiments (DoE) framework, implemented via Design-Expert software (version 11.0.0, Stat-Ease Inc., USA). A BBD was employed to evaluate both linear and quadratic effects of formulation variables, with each factor tested at three coded levels: low (−1), medium (0), and high (+1). This design includes points at the midpoints of edges and the center of the experimental space, excluding corner points to improve efficiency and reduce the number of required experiments.

Based on preliminary trials and literature data, the concentrations of Tween 80, Tween 20, and sodium alginate (%) were selected as independent variables, while particle size, zeta potential (ζ-potential), and polydispersity index (PDI) were chosen as dependent variables (responses).

A total of 14 experimental runs were performed, consisting of 12 unique formulations and 2 replicates at the center point to assess reproducibility and experimental error (see Table 3 and 4). The experimental data were fitted to a second-order polynomial model for response prediction, as described by eq 1:

1 $$Y=\beta_{0}+\beta_{1}X_{1}+\beta_{2}X_{2}+\beta_{3}X_{3}+\beta_{12}X_{1}X_{2}+\beta_{13}X_{1}X_{3}+\beta_{23}X_{2}X_{3}+\beta_{11}{X_{1}}^{2}+\beta_{22}{X_{2}}^{2}+\beta_{33}{X_{3}}^{2}$$

where *Y* is the predicted response, *β*_*0*_ is the intercept, *β*_*1*_ to *β*_*33*_ are the regression coefficients, and *X*_*1*_, *X*_*2*_, *X*_*3*_ are the coded levels of the independent variables, including interaction and quadratic terms.^24,27,38^

### 2.4 Physicochemical Characterization of the Essential Oil Nanoemulsions

#### 2.4.1 Determination of Particle Size, Polydispersity Index, and Zeta Potential

Particle size and PDI were measured using Dynamic Light Scattering (DLS), while ζ was determined by electrophoretic mobility analysis. All measurements were conducted at 25 °C using a Zetasizer Nano ZS (ZEN3600 Malvern Instruments Ltd., UK). The analyses included formulations developed through BBD as well as the optimized EO nanoemulsions. Prior to measurement, samples were diluted 1:10 with ultrapure water to avoid multiple scattering effects. Each sample was analyzed in triplicate, and results are presented as mean ± standard deviation.

#### 2.4.2 FTIR Analysis of Essential Oils and Optimized Nanoemulsion Formulations

The compatibility between the active components and excipients in the nanoemulsion formulations was assessed using FTIR (Fourier Transform Infrared) spectroscopy. FTIR spectra were recorded for *E. citriodora* and *E. radiata* EOs, and their corresponding nanoemulsions, with examinations conducted over the spectral range of 4000–400 cm^–1^ (Shimadzu IRPrestige-12, Japan).

#### 2.4.3 Morphological Evaluation of Optimized Nanoemulsion Formulations

The surface morphology of the optimized EO nanoemulsions was characterized using scanning electron microscopy (SEM) (Carl Zeiss Ultra Plus, Germany). Sample preparation involved depositing a small aliquot of nanoemulsion onto a clean glass slide, followed by thorough air-drying to remove residual moisture. Subsequently, the dried samples were sputter-coated with a thin layer of gold to enhance electrical conductivity, enabling high-resolution imaging. SEM observations provided insights into the surface texture and structural features of the nanoemulsions, aiding in the assessment of formulation quality and droplet morphology.^39,40^

#### 2.4.4 Determination of Emulsion Type of Optimized Nanoemulsion Formulations

The type of emulsion was determined by measuring electrical conductivity due to the method’s high reliability and simplicity. Conductivity measurements were performed using a Thermo Scientific Orion Versa Star VSTAR9 conductivity meter equipped with a VSTAR-CBD Conductivity Module and an Orion Dura Probe 4-Cell Conductivity Electrode (USA). To examine the impact of temperature on conductivity, measurements were carried out at both ambient (25 °C) and physiological (37 °C) conditions.^41^

#### 2.4.5 Determination of pH of the Optimized Nanoemulsion Formulations

The pH of the optimal EO nanoemulsion formulations was measured using a pH meter (Ohaus Starter 3100, USA) equipped with a glass electrode. Prior to measurements, the pH meter was calibrated with standard buffer solutions. The pH electrode was directly immersed into the formulations for measurement. Each sample was analyzed in triplicate, and the results were reported as the mean ± SD.^27,42^

#### 2.4.6 Measurement of Viscosity of the Optimized Nanoemulsion Formulations

Viscosity measurements of the optimized EO nanoemulsions were performed using a Brookfield DV-I Prime Digital Viscometer (Brookfield Engineering Laboratories, Middleboro, MA, USA) operating at 50 rpm. The instrument was fitted with an LV-3 cone spindle (Brookfield Engineering Laboratories, Middleboro, MA, USA), selected to provide accurate viscosity readings for low-viscosity nanoemulsions. To maintain consistent and physiologically relevant thermal conditions, the samples were equilibrated in a thermostatically controlled water bath at ambient (25 °C) and physiological (37 °C) temperatures during all measurements. Measuring at these two temperatures simulates ambient storage and topical application conditions, respectively. Prior to testing, the samples were equilibrated within the instrument for 15 min to minimize transient flow effects and allow temperature equilibration. Each formulation was measured in triplicate, and the results were reported as mean ± SD.^43,44^

### 2.5 Stability Studies

#### 2.5.1 Evaluation of Long-Term Storage Stability

The physicochemical stability of the optimized nanoemulsions was evaluated over a 90-day period. Samples were stored in sealed vials, protected from light, within a stability chamber maintained at 25 °C and 60% relative humidity (RH) to simulate typical ambient storage conditions. Assessments were conducted on days 1, 30, 60, and 90, including visual inspections for appearance changes (e.g., phase separation, turbidity) and Dynamic Light Scattering (DLS) analysis using a Zetasizer Nano ZS (model ZEN 3600, Malvern Instruments Ltd., UK) to determine particle size, PDI, and ζ. All measurements were performed in triplicate to ensure reproducibility and reliability.

#### 2.5.2 Accelerated Stability Studies

A modified three-phase accelerated stability protocol was employed to evaluate the stability of the optimized *E. citriodora* nanoemulsion (Opt-ECNE) and the *E. radiata* nanoemulsion (Opt-ERNE). Stability was defined by the absence of significant visual changes, such as phase separation, creaming, or flocculation. Additionally, DLS parameters specifically particle size, PDI, and zeta potential were measured at the final stage to detect any significant physicochemical alterations. The protocol involved the following sequential steps:

- The optimal formulations were subjected to centrifugation at 3500 rpm for 30 min. Following this step, they were evaluated under heating/cooling stress conditions.
- Heating/Cooling Cycles: Samples underwent six alternating cycles between refrigerated conditions (4 °C) and elevated temperature (45 °C), with each temperature maintained for at least 48 h.
- Freeze/Thaw Cycles: The formulations were subjected to three freeze–thaw cycles, each consisting of 24 h at 20 °C followed by thawing at room temperature. Following these cycles, samples were centrifuged again and assessed for phase separation.^20,45^

### 2.6 Antibacterial and Antibiofilm Activity Tests

#### 2.6.1 Preparation of the Essential Oils, Nanoemulsions, and Bacteria

After preparing the stock solutions of the *E. citriodora* and *E. radiata* EOs and their nanoemulsions in dimethyl sulfoxide (DMSO), the final concentration of DMSO was adjusted to 3% using Mueller Hinton broth (MHB, Liofilchem, Italy). The final concentrations of the EOs and nanoemulsions ranged from 150 to 1.17 mg/mL.

Antibacterial and antibiofilm activities of the EOs, Opt-ECNE, and Opt-ERNE were evaluated against *Staphylococcus aureus* ATCC 25923, *Enterococcus faecalis* ATCC 29212, and *Klebsiella pneumoniae* ATCC 700603. A fresh pure culture of each strain on Mueller Hinton agar (MHA, Liofilchem, Italy) was used to prepare an inoculum of 1.5 × 10^8^ colony forming unit (CFU) per mL in MHB.

#### 2.6.2 Antibacterial Activity Test

Minimum inhibitory concentrations (MICs) of the EOs, Opt-ECNE, and Opt-ERNE against the tested bacteria were evaluated using the microdilution method (CLSI, 2023).^46^ 100 μL of the 2-fold serial dilutions of the EOs and the optimized nanoemulsions prepared in 96-well microplates using MHB were mixed with each of the bacteria. The final concentrations of the bacteria in the wells were 5 × 10^5^ CFU/mL. The microplates were incubated at 37 °C under an aerobic atmosphere for 18 h. MICs were regarded as the minimum concentration of the EOs and the nanoemulsions that visually stopped the growth of bacteria. After macroscopical examination, 10 μL of 5 mg/mL 3-[4,5-dimethylthiazol-2-yl]-2,5-diphenyltetrazolium bromide (MTT) was added to the wells. The smallest concentration of the EOs and nanoemulsions at which there is no change in the color of MTT to purple was confirmed as the MIC. Ciprofloxacin was used as the standard antibacterial agent. The highest concentrations of the EOs and the nanoemulsions were used as negative control, whereas the bacteria inoculated in wells containing MHB alone were used as positive control. Emulsions without the EOs (distilled water used instead of the oil) were also tested by the microdilution test. The tests were done in triplicate.

#### 2.6.3 Biofilm Inhibition Assay

Inhibition of biofilm formation by the EOs, Opt-ECNE, and Opt-ERNE was investigated using a crystal violet staining-based technique as suggested by Haney et al.^47^ A single bacterial colony inoculated in MHB was incubated in a shaking water bath under an aerobic atmosphere at 37 °C for 16–20 h. Twofold serial dilutions of sub-MIC concentrations of the EOs and the nanoemulsions were mixed with the bacteria in a 96-well microtiter plate. The plates were incubated overnight at 37 °C under an aerobic atmosphere. After rinsing the wells with distilled water three times, 0.1% crystal violet dye was added to each well. Following incubation at room temperature for 30 min, the wells were washed three times with distilled water and treated with 70% ethanol solution. Following 30 min destaining with the alcohol, absorbance of each well was recorded at 595 nm using a spectrophotometer (Thermo Scientific Varioskan Flash, USA). The percentage of biofilm growth was calculated by comparing the absorbances of the wells containing the EO and the optimized nanoemulsions with those of growth control (wells containing bacteria without the EO and the optimized nanoemulsion) and sterility control that includes sub-MIC concentrations of the EOs and nanoemulsions without the bacteria.

### 2.7 Statistical Methods and Data Analysis

For formulation studies, data were analyzed using regression and one-way analysis of variance (ANOVA) conducted with Design-Expert software (Version 11.0.0). Data obtained from experimental studies were further evaluated by one-way ANOVA using GraphPad Prism (Version 6.0.1). Multiple group comparisons were performed using Tukey’s and Dunnett’s multiple comparison tests. Statistical significance was established at a *p*-value <0.05.

Antibacterial activity tests and biofilm inhibition assays were carried out in triplicate. The data were assessed using the means ± standard error of mean (SEM). Student’s *t* test in Excel and Prism version 10.4.0 was used to determine the statistical significance.

## 3 Results and Discussion

### 3.1 Determination of the Composition of Essential Oils

Three *Eucalyptus* EO compositions were analyzed and confirmed by 83.4–99.6% relative content, overall. The identified compounds, along with their relative percentages (%), RRIs calculated in the present study and RRIs reported in the literature, are listed in Table 2.

**Table 2:** Volatile Compounds in *Eucalyptus* Essential Oils

RRI^a^	RRI^b^	Compound	*E. citriodora* (%)^c^	*E. globulus* (%)^c^	*E. radiata* (%)^c^	Literature
1032	1032	α-Pinene	–	11.4	–	^48^
1076	1076	Camphene	–	0.1	–	^48^
1118	1118	β-Pinene	–	11.4	0.2	^48^
1174	1174	Myrcene	0.4	–	–	^49^
1176	1186	α-Phellandrene	–	4.8	–	^50^
1203	1203	Limonene	–	5	2.8	^50^
1213	1213	1,8-Cineole	0.4	30.9	93.1	^50^
1255	1256	γ-Terpinene	0.7	4.5	0.1	^50^
1280	1278	*p*-Cymene	–	10	1	^50^
1290	1261–1300	Terpinolene	–	0.4	–	^51^
1487	1487	Citronellal	79.9	–	–	^52^
1497	1497	α- Copaene	–	0.1	–	^53^
1544	1544	α-Gurjunene	–	0.1	–	^53^
1553	1553	Linalool	–	tr^d^	0.6	^31^
1586	1545–1600	Pinocarvone	–	tr^d^	–	^54^
1592	1592	Fenchyl alcohol	0.1	0.3	–	^55^
1611	1611	Terpinen-4-ol	–	1.8	0.2	^53^
1612	1612	β-Caryophyllene	0.3	0.7	–	^45^
1628	1628	Aromadendrene	0.4	1.4	–	^50^
1661	1661	Alloaromadendrene	–	0.3	–	^53^
1670	1670	*trans*-Pinocarveol	–	0.4	0.2	^56^
1682	1682	δ-Terpineol	0.2	tr^d^	–	^57^
1687	1687	α-Humulene	0.2	0.2	–	^49^
1700	1700	Limonen-4-ol	–	0.3	–	^33^
1706	1706	α-Terpineol	–	3.1	1.2	^49^
1709	1709	α-Terpinyl acetate	–	0.8	–	^58^
1755	1755	Bicyclogermacrene	0.2	0.2	–	^59^
1776	1776	δ-Cadinene	0.3	0.3	–	^31^
1804	1804	Myrtenol	–	tr^d^	–	^60^
2250	2250	α-Eudesmol	–	0.9	–	^58^
2257	2258	β-Eudesmol	0.3	1.3	0.2	^50^
		TOTAL	83.4	90.7	99.6	

a Relative retention indice of the compound calculated against *n*-alkanes (C_9_–C_40_) on HP-Innowax column.

b Relative retention indice of the compound on polar column reported in the literature.

c Mean % calculated from FID data.

d tr = trace (<0.1%).

In the current study, the major constituents of *E. globulus* EO were identified as 1,8-cineole (30.9%), α-pinene (11.4%), and β-pinene (11.4%). Citronellal (79.9%) was identified as the dominant component of *E. citriodora* EO, whereas 1,8-cineole (93.1%) was determined to be the predominant constituent of *E. radiata* EO. In previous studies, the 1,8-cineol content of *E. globulus* EO was reported to exceed 50%^61^ while the citronellal in *E. citriodora* essential oil ranged between 50% to 70%.^62–64^ Additionally, the relative amount of 1,8-cineole in *E. radiata* was reported to surpass 60%.^65^ The major terpenoids and other EO constituents identified in the present study, as detailed in Table 2, are in agreement with the previously published data.

### 3.2 Evaluation of the Box–Behnken Design for *E. globulus*, *E. citriodora*, and *E. radiata* Essential Oil Formulations

Among the *Eucalyptus globulus*, *E. citriodora*, and *E. radiata* EOs initially evaluated for nanoemulsion development, *E. globulus* showed notable formulation difficulties. Preliminary trials consistently failed to yield physically stable nanoemulsions with *E. globulus*, likely due to unfavorable interactions between its constituents and the emulsifier system. *E. globulus* EO is particularly rich in monoterpenes predominantly α- and β-pinene (>10%) (as in Table 2) which are highly hydrophobic and of low viscosity. These components can insert between surfactant molecules at the oil–water interface, disturbing interfacial packing and weakening film cohesion. Moreover, the positional isomerism of *α-* and *β-*pinene alters their polarity and affinity toward the surfactant monolayer, reducing interfacial stability and promoting droplet coalescence. Consequently, effective reduction of interfacial tension could not be achieved, resulting in rapid phase separation.^61,66,67^

Following the successful emulsification of *E. citriodora* and *E. radiata* EOs, the optimization of eucalyptus oil nanoemulsions was accomplished using a BBD within the response surface methodology framework. As presented in Tables 3 and 4, a total of 14 distinct formulations (12 unique formulations with 2 center-point replicates) were generated via Design-Expert software to systematically evaluate the effects of surfactant and stabilizer concentrations on droplet size, ζ-potential, and PDI.

**Table 3:** Box–Behnken Design Results for *E. citriodora* Nanoemulsions, Showing the Influence of Independent Variables (Tween 80, Tween 20, and Sodium Alginate Solution (%)) over Responses (Particle Size, ζ-Potential, and Polydispersity Index)

Batch No	Factor*X*_1_: Tween 80 (mg)	Factor*X*_2_: Tween 20 (mg)	Factor*X*_3_: Sodium alginate sol. (% q.s. 2000 mg)	Response*Y*_1_: Particle Size (nm)	Response*Y*_2_: Zeta Potential (mV)	Response*Y*_3_: Polydispersity Index (PDI)
1	100	30	0.10	175.00	–17.20	0.227
2	100	10	1.00	229.50	–22.90	0.459
3	150	30	0.55	172.80	–21.10	0.21
4	150	10	0.55	187.30	–22.20	0.253
5	100	20	0.55	215.20	–18.40	0.235
6	50	20	0.10	262.50	–13.30	0.363
7	150	20	0.10	176.20	–17.70	0.163
8	100	10	0.10	197.60	–14.10	0.251
9	100	30	1.00	221.70	–23.30	0.412
10	150	20	1.00	213.90	–25.30	0.353
11	50	10	0.55	256.30	–18.60	0.327
12	50	30	0.55	252.90	–19.50	0.289
13	50	20	1.00	270.10	–22.00	0.523
14	100	20	0.55	192.80	–19.70	0.285

**Table 4:** Box–Behnken Design Results for *E. radiata* Nano-Emulsions, Showing the Influence of Independent Variables (Tween 80, Tween 20, and Sodium Alginate Solution (%)) over Responses (Particle Size, ζ-Potential, and Polydispersity Index)

Batch No	Factor*X*_1_: Tween 80 (mg)	Factor*X*_2_:Tween 20 (mg)	Factor*X*_3_: Sodium alginate solution (% q.s. 2000 mg)	Response*Y*_1_: Particle Size (nm)	Response*Y*_2_: Zeta Potential (mV)	Response*Y*_3_: Polydispersity Index (PDI)
1	100	30	0.10	270.60	–21.00	0.350
2	100	10	1.00	275.20	–19.10	0.312
3	150	30	0.55	205.00	–18.10	0.194
4	150	10	0.55	182.70	–19.30	0.130
5	100	20	0.55	239.00	–21.50	0.340
6	50	20	0.10	382.10	–18.30	0.402
7	150	20	0.10	390.00	–31.20	0.460
8	100	10	0.10	210.20	–16.70	0.206
9	100	30	1.00	228.50	–21.00	0.265
10	150	20	1.00	130.50	–11.00	0.164
11	50	10	0.55	262.40	–15.10	0.210
12	50	30	0.55	280.10	–14.30	0.180
13	50	20	1.00	326.70	–20.80	0.323
14	100	20	0.55	215.80	–15.00	0.282

A blend of Tween 20 and Tween 80 proved superior to Span 80, effectively minimizing interfacial tension and improving stability,^37^ while sodium alginate enhanced uniformity. Statistical analysis confirmed the model’s reliability (R^2^ > 0.95, *p* < 0.05), and response surface plots revealed significant interactions between formulation variables. This systematic approach enabled precise fine-tuning of composition (Figures 2 and 3), resulting in stable and homogeneous nanoemulsions suitable for topical application.^24,25^

**Figure 2:**
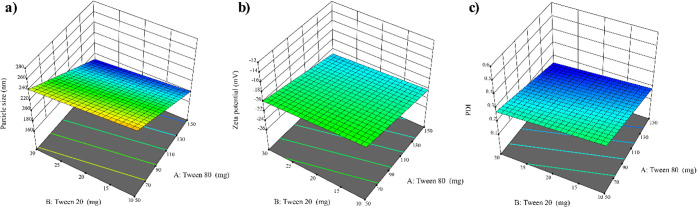
3D surface plots showing the influence of Tween 80 and Tween 20 concentrations at a fixed sodium alginate level (0.1% w/v) on the physicochemical properties of *E. citriodora* nanoemulsions: (a) Particle size (nm), (b) ζ-potential (mV), (c) PDI.

**Figure 3:**
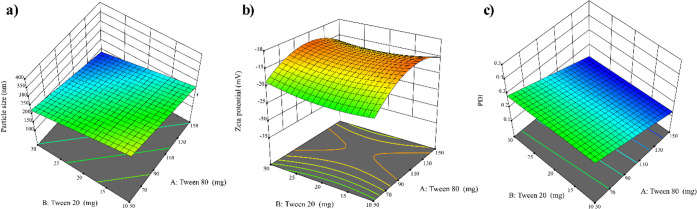
3D surface plots showing the influence of Tween 80 and Tween 20 concentrations at a fixed sodium alginate level (0.1% w/v) on the physicochemical properties of *E. radiata* nanoemulsions: (a) Particle size (nm), (b) ζ-potential (mV), (c) PDI.

The selection of Tween 20 (HLB 16.7) and Tween 80 (HLB 15.0) was guided by their excellent biocompatibility, stability, and established use in pharmaceutical, cosmetic, and food formulations. Their complementary HLB values facilitated efficient oil-in-water emulsification and the formation of stable, nanosized droplets. Tween 20 rapidly adsorbed at the interface to lower interfacial tension, whereas Tween 80 contributed to enhanced droplet stabilization and reduced irritation potential.^68–70^ Sodium alginate, incorporated as a natural stabilizer, increased viscosity and provided steric protection, minimizing droplet coalescence and Ostwald ripening. Its interaction with surfactants further reinforced the interfacial film, ensuring consistent drug release and sustained therapeutic efficacy in infection-targeted applications. Collectively, these components acted synergistically to produce a stable and biocompatible nanoemulsion matrix suitable for topical drug delivery.^71–73^

ANOVA confirmed that the fitted polynomial model significantly supported the variation in droplet size (*Y*_*1*_ *)* for *E. citriodora* nanoemulsions (F = 18.74, *p* = 0.0002; eq 2; Table 5; Figure 2a). The model exhibited satisfactory goodness-of-fit (R^2^ = 0.8490; Adj R^2^ = 0.8037; Pred R^2^ = 0.7081) and a nonsignificant lack-of-fit (*p* = 0.6806), supporting its adequacy for predicting droplet size within the studied design space.

**Table 5:** Optimization Parameters and Response Variables for *E. citriodora* and *E. radiata* Nano-Emulsions

		Level of variables	
Factors—Independent variables	Units	Coded Low (−1)	Coded high (+1)
*X*_1_ = Tween 80	mg	50.00	150.00
*X*_2_ = Tween 20	mg	10.00	30.00
*X*_3_ = Alginic acid solution (q.s. 2000 mg)	%	0.10	1.00

Responses—Depended variables	Units	Constrains
*Y*_1_ = Particle size	nm	Minimize
*Y*_2_ = Zeta Potential	mV	Minimize
*Y*_3_ = PDI	-	Minimize

Regression equations of fitted models for *E. citriodora* nanoemulsions
2 $$Y_{1}=282.10-72.69X_{1}-0.6X_{2}+34.41X_{3}$$
3 $$Y_{2}=-10.84-0.03X_{1-}0.04X_{2}-8.67X_{3}$$
4 $$Y_{3}=0.34-0.001X_{1}-0.02X_{2}+0.21X_{3}$$

Regression equations of fitted models for *E. radiata* nanoemulsions
5 $$Y_{1}=369.73-0.91.X_{1}-4.42X_{2}+122.50X_{3}$$
6 $$Y_{2}=-34.10+0.36X_{1}-0.19X_{2}+6.17X_{3}-0.01X_{1}X_{2}+0.11X_{1}X_{3}-0.44X_{2}X_{3}-0.02{X_{1}}^{2}+0.013{X_{2}}^{2}-14.19{X_{3}}^{2}$$
7 $$Y_{3}=0.35-0.02X_{1}-0.01X_{2}+0.18X_{3}$$

Among the studied variables, Tween 80 concentration (*X*_*1*_) exerted the most significant influence on droplet size reduction (*p* < 0.0001), reflecting its superior interfacial activity and stabilizing capability. In contrast, Tween 20 (*X*_*2*_) exhibited no statistically significant effect within the investigated range (*p* = 0.28).^74,75^ Sodium alginate (*X*_*3*_), however, showed a positive correlation with droplet size (*p* = 0.016), likely attributable to increased viscosity of the continuous phase that limits droplet disruption and promotes coalescence. Response surface analysis (Figure 2a) further illustrates these relationships, defining the optimal surfactant: Polymer ratio required to achieve minimal droplet size and enhanced formulation stability size.^25,76^

ANOVA confirmed that the linear model significantly described the variation in ζ-potential (*Y*_*2*_ *)* for *E. citriodora* nanoemulsions (F = 49.38, *p* < 0.0001; eq 3; Table 5; Figure 2b). The model showed strong goodness-of-fit (R^2^ = 0.9368; Adj R^2^ = 0.9178; Pred R^2^ = 0.8732; Adeq Precision = 20.9320) and a nonsignificant lack-of-fit (*p* = 0.6215), supporting its adequacy within the studied design space. Sodium alginate (*X*_*3*_) had the most pronounced effect (*p* < 0.001), significantly increasing ζ-potential and promoting electrostatic stabilization. Tween 80 (*X*_*1*_) also contributed positively, whereas Tween 20 (*X*_*2*_) had no significant influence within the tested range. Three-dimensional response surface plots (Figure 2b) further illustrate these interactions, highlighting the critical roles of sodium alginate and Tween 80 in maximizing ζ-potential and ensuring nanoemulsion stability.^72,77^

The PDI (*Y*_*3*_ *),* which reflects droplet size distribution uniformity (0 = highly uniform; 1 = broadly dispersed),^78^ was adequately described by a statistically significant linear model for *E. citriodora* nanoemulsions (F = 12.30, *p* = 0.0011; eq 4; Table 5; Figure 2c). The model showed acceptable fit statistics (R^2^ = 0.7868; Adj R^2^ = 0.7228; Pred R^2^ = 0.5850; Adeq Precision = 11.0435) and a nonsignificant lack-of-fit (*p* = 0.4616), supporting its suitability within the investigated design space. Among the formulation variables, Tween 80 (*X*_*1*_) and sodium alginate (*X*_*3*_) significantly influenced PDI (*p* = 0.0062 and *p* < 0.001, respectively), whereas Tween 20 (*X*_*2*_) had no significant effect within the studied range (*p* = 0.34).

Formulations with PDI values below 0.25 demonstrated superior colloidal stability, as higher surfactant concentrations promoted the formation of more uniform nanoemulsions. The strong interfacial adsorption of Tween 80 contributes to the formation of a compact steric barrier, effectively preventing droplet coalescence and flocculation. In the presence of sodium alginate, additional multilayered interfacial structures likely form, further reinforcing mechanical and colloidal stability. This cooperative steric stabilization remains robust under varying ionic conditions, enabling the generation of stable, monodisperse *E. citriodora* nanoemulsions.^22,24,79^

For *E. radiata* nanoemulsions, droplet size (*Y*_*1*_ *)* was best described by a significant linear model (F = 16.04, *p* = 0.0004; eq 5; Table 5; Figure 3a), with a nonsignificant lack-of-fit (*p* = 0.6357), supporting model adequacy within the investigated design space. The model showed acceptable goodness-of-fit (R^2^ = 0.8280; Adj R^2^ = 0.7763; Pred R^2^ = 0.6519; Adeq Precision = 10.9954). All three formulation variables significantly affected *Y*_*1*_ (Tween 80: *p* = 0.0037; Tween 20: *p* = 0.0045; sodium alginate: *p* = 0.0011), consistent with the fitted equation (Table 5; eq 5), with response surface analysis (Figure 3a) showing minimum droplet sizes at higher surfactant concentrations.

Statistical evaluation confirmed the model’s robustness (*p* < 0.001), consistent with literature indicating kinetically stable colloidal systems with droplet sizes of 20–200 nm enhances solubility and stability of lipophilic compounds.^200^ These trends align with previous eucalyptus oil nanoemulsion studies, where increased surfactant levels reduced droplet size and polysaccharide stabilizers improved stability via viscosity enhancement and steric effects.^28,80^

For *E. radiata* nanoemulsions, ζ-potential (*Y*_*2*_) was best described by a significant quadratic model (*p* = 0.0065; eq 6; Table 5), with good goodness-of-fit (R^2^ = 0.9764; Adj R^2^ = 0.9232; Pred R^2^ = 0.6217; Adeq Precision = 17.3060). Tween 80 (*X*_*1*_) and sodium alginate (*X*_*3*_) showed significant main effects (*p* = 0.0066 and *p* = 0.0021, respectively), whereas Tween 20 alone was not significant (*p* = 0.4601). In addition, the interaction terms *X*_*1*_ *X*_*3*_ and *X*_*2*_ *X*_*3*_ (*p* = 0.0183 and *p* = 0.0370, respectively) and the quadratic terms *X*_*1*_^*2*^ and *X*_*3*_^*2*^ (*p* = 0.0031 and *p* = 0.0167, respectively) were significant, indicating a nonlinear, composition-dependent influence of surfactant–polymer interactions on interfacial charge behavior. Sodium alginate initially increased negative ζ-potential via dissociated carboxylate groups, whereas nonlinear interactions with surfactants modulated this effect. Tween 80 contributed through interfacial adsorption and formation of a stabilizing film. Maximum negative *ζ-*potential values (−25 to −30 mV) occurred at low sodium alginate, high Tween 80, and low Tween 20 concentrations (Figure 3b), highlighting the complex interaction of polymer and surfactant in electrostatic stabilization.^72,73,81,82^

For *E. radiata* nanoemulsions, PDI (*Y*_*3*_ *)* was effectively described by a significant linear model (F = 34.90, *p* < 0.0001; eq 7; Table 5), with strong goodness-of-fit (R^2^ = 0.9128; Adj R^2^ = 0.8867; Pred R^2^ = 0.8440; Adeq Precision = 19.0983) and a nonsignificant lack-of-fit (*p* = 0.8775), supporting model adequacy within the investigated design space. Among the variables, Tween 80 (*X*_*1*_ *)* and sodium alginate (*X*_*3*_ *)* significantly influenced PDI (*p* < 0.0001 for both), whereas Tween 20 (*X*_*2*_ *)* showed no significant effect within the studied range (*p* = 0.5099), with optimal values (∼0.2–0.3) achieved at higher Tween 80 and lower Tween 20 concentrations. Tween 80 improved droplet uniformity, while sodium alginate slightly increased PDI, likely due to viscosity-induced droplet bridging. In comparison, *E. citriodora* required higher levels of both surfactants to achieve similar monodispersity, reflecting differences in interfacial behavior driven by EO composition and polarity.^83,84^

The optimization of nanoemulsion formulations was guided by predefined target criteria (constraints) summarized in Table 5, defining the optimal design space. Contour plots (Figures 4 and 5) illustrated the critical combinations of formulation factors necessary to achieve the desired outcomes. The desirability function (d), a dimensionless index ranging from 0 to 1, was employed to identify the most favorable formulations, with a value of 1 representing perfect alignment with all target responses.^85^

**Figure 4:**
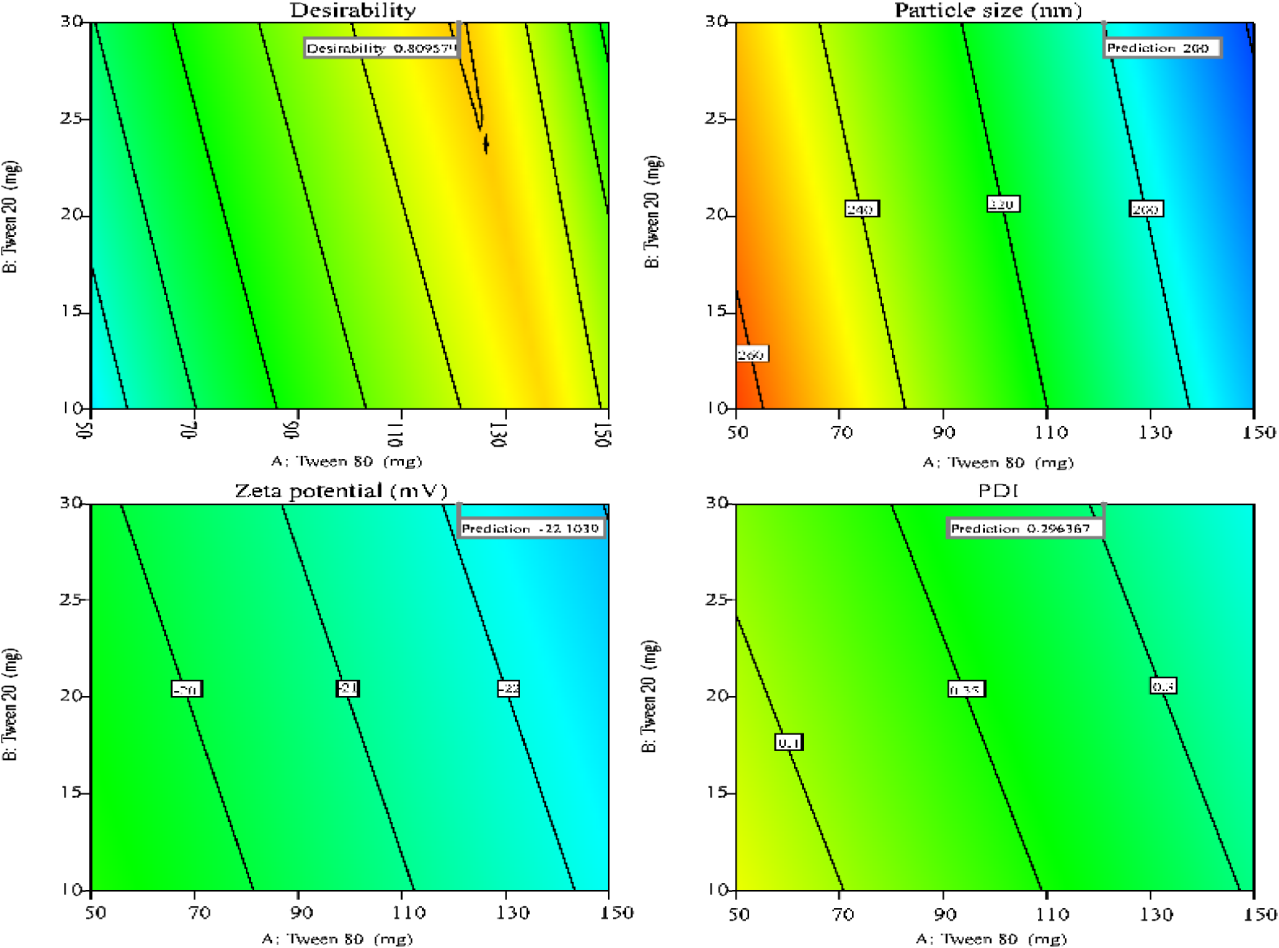
Desirability plots depicting the optimal formulation conditions and design range regions where particle size, ζ-potential, and PDI are optimized according to predefined criteria for *E. citriodora* nanoemulsions.

**Figure 5:**
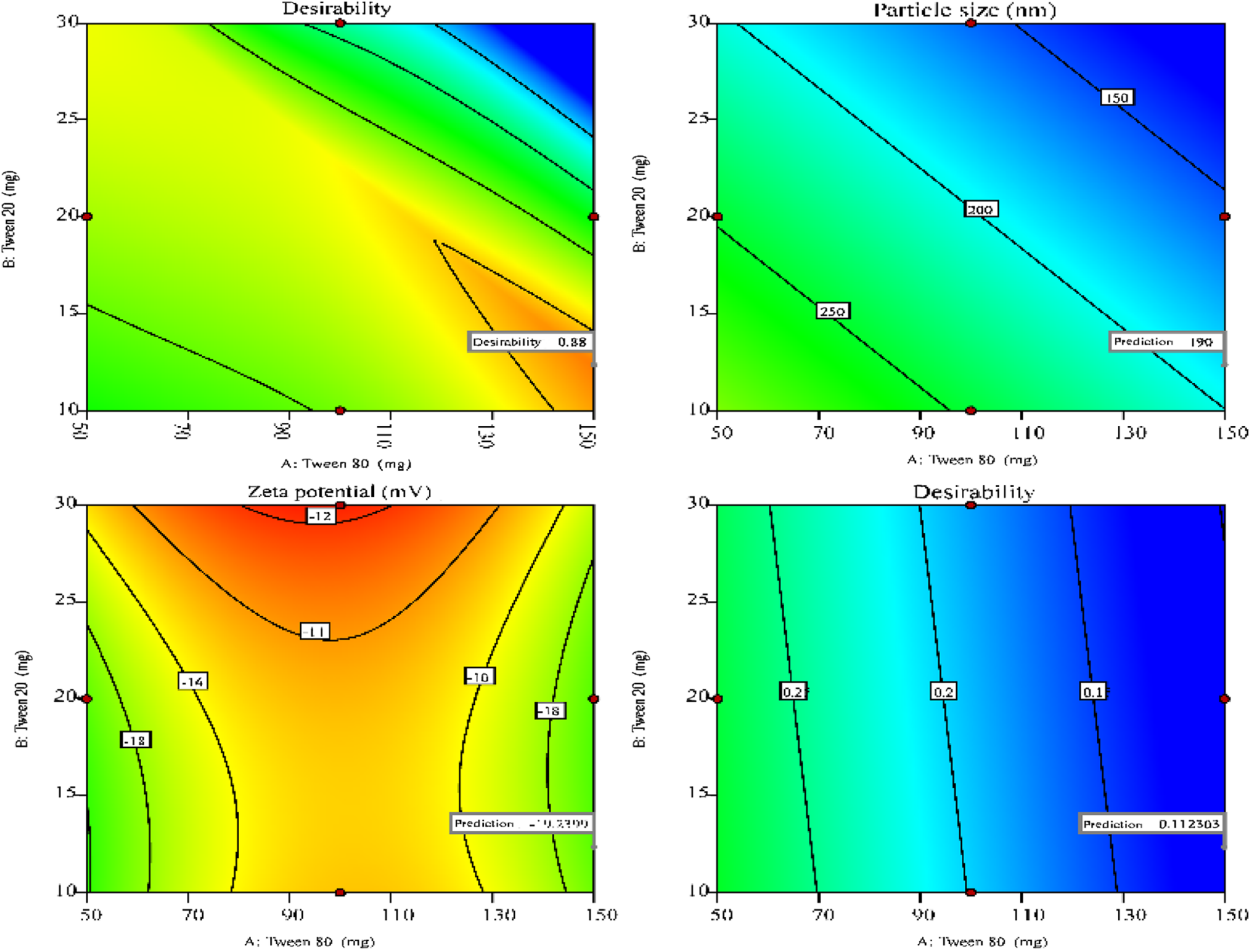
Desirability plots depicting the optimal formulation conditions and design range regions where particle size, ζ-potential, and PDI are optimized according to predefined criteria for *E. radiata* nanoemulsions.

Based on desirability values, two formulations were selected as optimal: Opt-ECNE (*E. citriodora*) with d = 0.82 and Opt-ERNE (*E. radiata*) with d = 0.88 (Table 6). Experimental evaluation of these formulations (Table 6; Figure 6) confirmed that measured properties closely matched model predictions, falling within the 95% confidence intervals. These results validate the robustness and predictive reliability of the statistical optimization approach employed.

**Figure 6:**
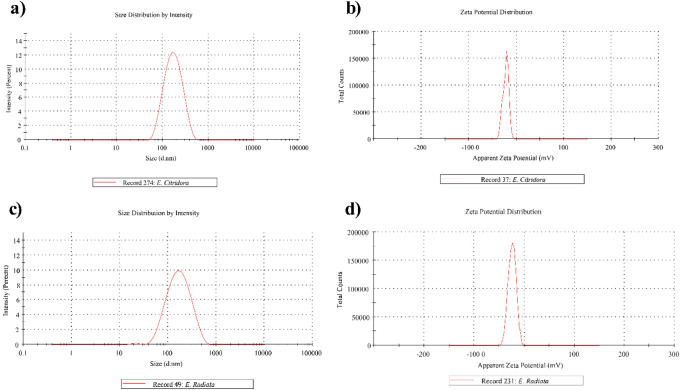
Zetasizer analysis of the optimized EO nanoemulsions. (a) Particle size distribution of the Opt-ECNE, (b) Average zeta potential distribution of Opt-ECNE, (c) Particle size distribution of the Opt-ERNE, (d) Average zeta potential distribution of the Opt-ERNE.

**Table 6:** Model Validation with Selected Optimized *E. citriodora* and *E. radiata* Nanoemulsions

Formulation Code	Opt-ECNE	Opt-ERNE
Run	**1**	**2**
*X1*: Tween 80 (mg)	150.00	150.00
*X2:* Tween 20 (mg)	10.00	12.35
*X3*: Sodium Alginate Solution (%; q.s. 2000 mg)	0.29	0.10
Desirability Value	0.82	0.88
*Y1*: Particle size (nm)		
Observed responses	186.50 ± 0.50	190.30 ± 0.30
95% CI Low for Mean	164.64	141.95
95% CI High for Mean	194.49	238.05
*Y2: ζ*-Potential (mV)		
Observed responses	–22.00	–22.90
95% CI Low for Mean	–22.24	–22.98
95% CI High for Mean	–20.30	–20.30
*Y3*: PDI		
Observed responses	0.245	0.227
95% CI Low for Mean	0.192	0.160
95% CI High for Mean	0.298	0.240

### 3.3 FTIR Characterization of Optimal Nanoemulsions

FTIR spectroscopy was conducted to assess the structural integrity and chemical compatibility of *E. citriodora* and *E. radiata* EOs within their respective nanoemulsions (Opt-ECNE and Opt-ERNE) (Figure 7).

**Figure 7:**
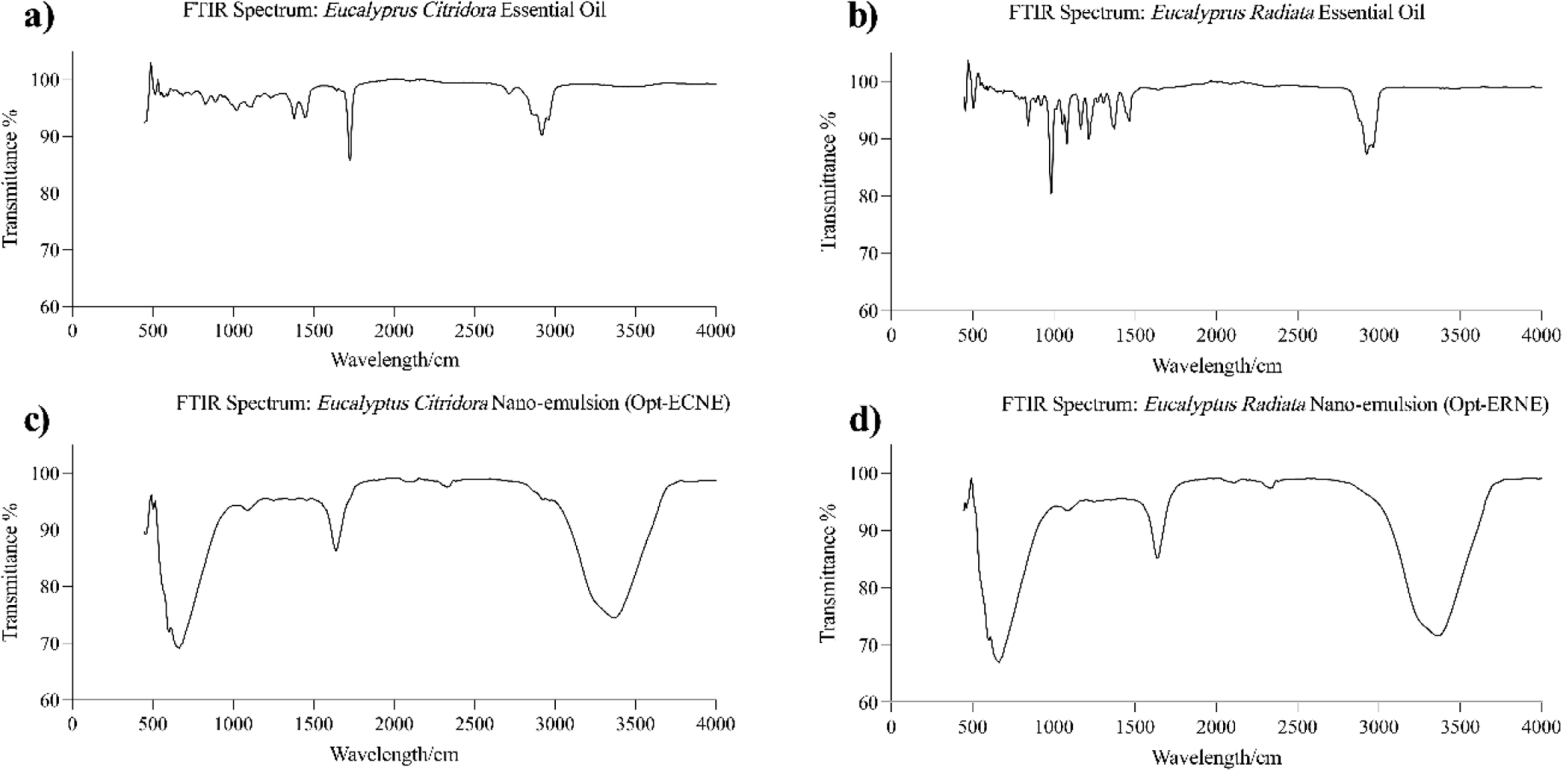
FTIR spectra of (a)*E. citriodora* essential oil, (b) Optimized *E. citriodora* nanoemulsion (Opt-ECNE), (c) *E. radiata* essential oil, and (d) Optimized *E. radiata* nanoemulsion (Opt-ERNE).

The IR spectrum of *E. citriodora* oil exhibited characteristic absorption bands indicative of its chemical composition. C–H stretching vibrations of aliphatic hydrocarbons appeared at 2960–2850 cm^–1^, while a prominent peak near 1735 cm^–1^ corresponded to the C=O stretching of aldehydes, primarily citronellal, the main bioactive constituent. The 1300–1500 cm^–1^ region reflected C–H bending vibrations from methyl and methylene groups, alongside overlapping modes of C–C and C–O bonds in monoterpenoids. Bands at 1100–1000 cm^–1^ indicated C–O stretching from alcohol or ether groups, such as citronellol, and absorptions below 900 cm^–1^ arose from terpene ring structures and fingerprint vibrations.^84,86,87^ In the Opt-ECNE, a broad O–H stretching band at 3300–3400 cm^–1^ suggested extensive hydrogen bonding between EO constituents and hydrophilic excipients (Tween 20, sodium alginate), enhancing physical stability. C–H stretching vibrations near 2900 cm^–1^ were retained, while the carbonyl peak shifted to ∼1650 cm^–1^, reflecting interactions within the nanoemulsion matrix without degradation of bioactive moieties.^73^

Similarly, *E. radiata* oil displayed C–H stretching bands at 2960–2850 cm^–1^ and a C=O stretching peak near 1735 cm^–1^ from ester or aldehyde groups. Strong C–O–C absorption between 1100–1000 cm^–1^ corresponded to 1,8-cineole, its major bioactive component.^67,85^ In Opt-ERNE, a broad O–H stretching band around 3400 cm^–1^ indicated hydrogen bonding with hydrophilic excipients, contributing to emulsion stability.^73^ Persistence of C–H and C=O functional groups, with minor intensity shifts in the fingerprint region, confirmed successful encapsulation and molecular dispersion without chemical degradation.^88,89^ Overall, FTIR analysis confirmed the successful incorporation of *E. citriodora* and *E. radiata* EOs into their nanoemulsions, preserving the chemical integrity of key functional groups and demonstrating structural stability and compatibility, supporting the sustained biological activity of the encapsulated oils.

### 3.4 Examination of the Morphology of the Essential Oil Nanoemulsions

SEM analysis (Figure 8) revealed that both Opt-ECNE and Opt-ERNE formulations displayed nearly spherical droplets with uniformly contoured, nonporous surfaces. The micrographs illustrated discrete and homogeneously distributed droplets with negligible agglomeration, reflecting the system’s colloidal stability and efficient surfactant coverage at the oil–water interface. The slight enlargement in apparent droplet size under SEM is commonly attributed to dehydration and film formation during sample preparation, which can induce partial coalescence or morphological deformation. The mean droplet diameters observed by SEM were in close agreement with those obtained by DLS, confirming the uniformity of the dispersion and the robustness of the emulsification process. Nevertheless, the strong correlation between SEM and DLS results supports the reproducibility of the nanoemulsion architecture and aligns with previous reports on stable surfactant-stabilized nanocarrier systems.^90–92^

**Figure 8:**
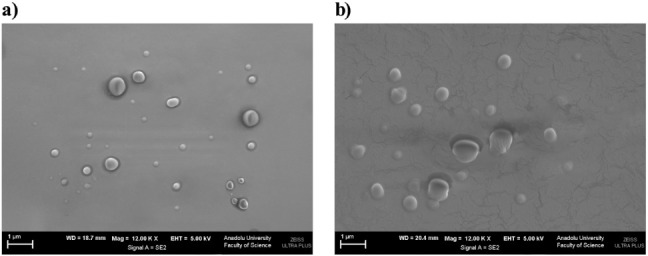
SEM micrographs of nanoemulsions: (a) Optimized *E. citriodora* nanoemulsion (Opt-ECNE) and (b) Optimized *E. radiata* nanoemulsion (Opt-ERNE), showing spherical droplets with uniform nanoscale size.

### 3.5 Determination of Emulsion Type

Conductivity measurements confirmed that all formulations were oil-in-water (O/W) nanoemulsions. At room temperature (∼25 °C), Opt-ECNE and Opt-ERNE exhibited high conductivity values of 276.00 ± 0.34 mS/cm and 363.40 ± 1.88 mS/cm, respectively, which remained similarly elevated at 37 °C (Opt-ECNE: 363.60 ± 0.12; Opt-ERNE: 441.00 ± 0.38), indicating stable dispersion of EO droplets within the aqueous phase. The measured conductivities align with typical O/W nanoemulsions (>10 mS/cm) and likely reflect differences in surfactant concentration, ionic strength, or oil composition. These results confirm that both nanoemulsions maintain a robust O/W structure across the tested temperatures.^93,94^

### 3.6 Determination of pH

The pH of a nanoemulsion critically affects its physicochemical stability, solubility, permeation, and the bioavailability of active components. For formulations targeting wound infections, pH also ensures compatibility with the skin barrier and minimizes irritation. In this study, Opt-ECNE and Opt-ERNE exhibited pH values of 5.8 ± 0.2 and 6.0 ± 0.4, respectively, falling within the physiological range of healthy human skin (4.5–6.5) and indicating suitability for topical application with minimal irritation risk.^95,96^

### 3.7 Viscosity Measurements of the Optimized Nanoemulsions

Viscosity is a key determinant of the physical stability, flow behavior, and topical performance of nanoemulsions. The optimized formulations exhibited viscosities of 21.00 ± 0.36 mPa·s (Opt-ECNE) and 18.00 ± 0.18 mPa·s (Opt-ERNE), characteristic of low-to-medium viscosity systems. This rheological profile promotes favorable spreadability, uniform film formation, and efficient percutaneous absorption. The obtained values align with those reported for O/W nanoemulsions of comparable composition. Viscosity in such systems is modulated by the dispersed-phase fraction, surfactant characteristics, droplet size, and processing parameters. Despite these influences, both formulations maintained stable viscosity throughout the study, confirming their structural integrity and suitability for dermal application.^44,97^

### 3.8 Stability Test

#### 3.8.1 Evaluation of Long-Term Storage Stability

Mean droplet size distribution and ζ-potential are key indicators of emulsion stability, reflecting susceptibility to destabilization mechanisms such as coalescence and flocculation; monitoring these parameters over time provides insight into structural integrity, with consistent values generally indicating prolonged stability.^98^ In this study, the optimized nanoemulsions, Opt-ECNE and Opt-ERNE, retained a uniform, opalescent appearance over 90 days, demonstrating effective stabilization by nanoscale droplet size and Brownian motion.^89,99^

Over 90 days, Opt-ECNE and Opt-ERNE exhibited minor but statistically significant increases in droplet size, from 186.50 ± 0.50 nm to 190.5 ± 0.56 nm (*p* < 0.001) and 190.30 ± 0.30 nm to 193.45 ± 0.23 nm (*p* < 0.001), respectively (Figure 9a), likely reflecting slight Ostwald ripening or droplet aggregation.^100,101^ ζ-Potential measurements indicated modest decreases, from −22.00 to −21.50 mV for Opt-ECNE (*p* < 0.05) and −22.90 to −21.70 mV for Opt-ERNE (*p* < 0.01) (Figure 9b), yet values remained below −21 mV, sufficient to maintain electrostatic repulsion and colloidal stability.^100^

**Figure 9:**
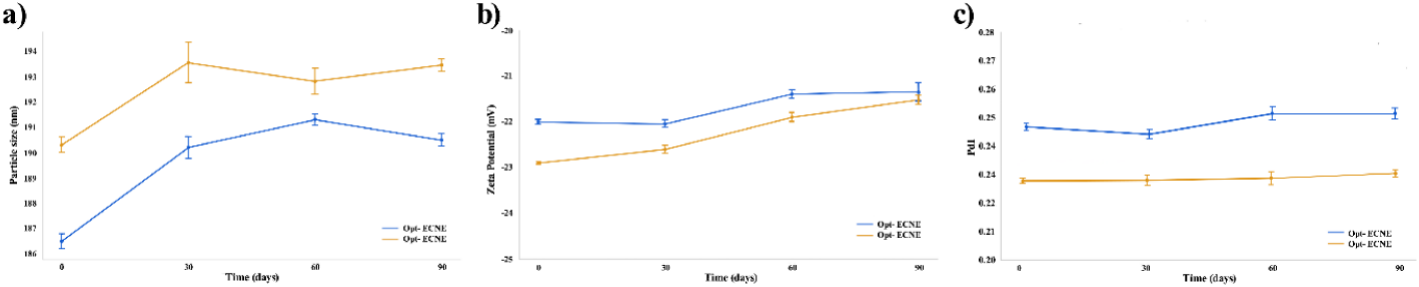
Long-term physical stability profiles of optimized nanoemulsions Opt-ECNE and Opt-ERNE monitored over 90 days (a) Particle size (Z-Average), (b) ζ-potential, (c) PDI.

PDI increased slightly over the same period (Opt-ECNE: 0.245 ± 0.005 to 0.248 ± 0.008; Opt-ERNE: 0.227 ± 0.004 to 0.229 ± 0.005; *p* < 0.05) (Figure 9c), but remained well below 0.3, indicating consistently narrow, uniform droplet distributions. Collectively, these results demonstrate that both nanoemulsions maintained excellent long-term physical and colloidal stability.^22^ Consistent with these findings, representative photographs documenting the unchanged macroscopic appearance of Opt-ECNE and Opt-ERNE over the 90-day storage period are provided in Supplementary Figure S1.

Although time-course chemical profiling was not performed in this study, the maintained nanoemulsion integrity and absence of macroscopic destabilization during storage suggest preservation of the encapsulated essential oils within the dispersed phase.^102,103^

#### 3.8.2 Accelerated Stability Test

Accelerated stability testing including centrifugation, heating–cooling, and freeze–thaw cycling was conducted to assess the thermodynamic robustness and long-term integrity of the optimized nanoemulsion systems. Both Opt-ECNE and Opt-ERNE maintained excellent physical stability during centrifugation and heating–cooling challenges, showing no visual evidence of creaming, cracking, or phase separation (Table 7).

**Table 7:**
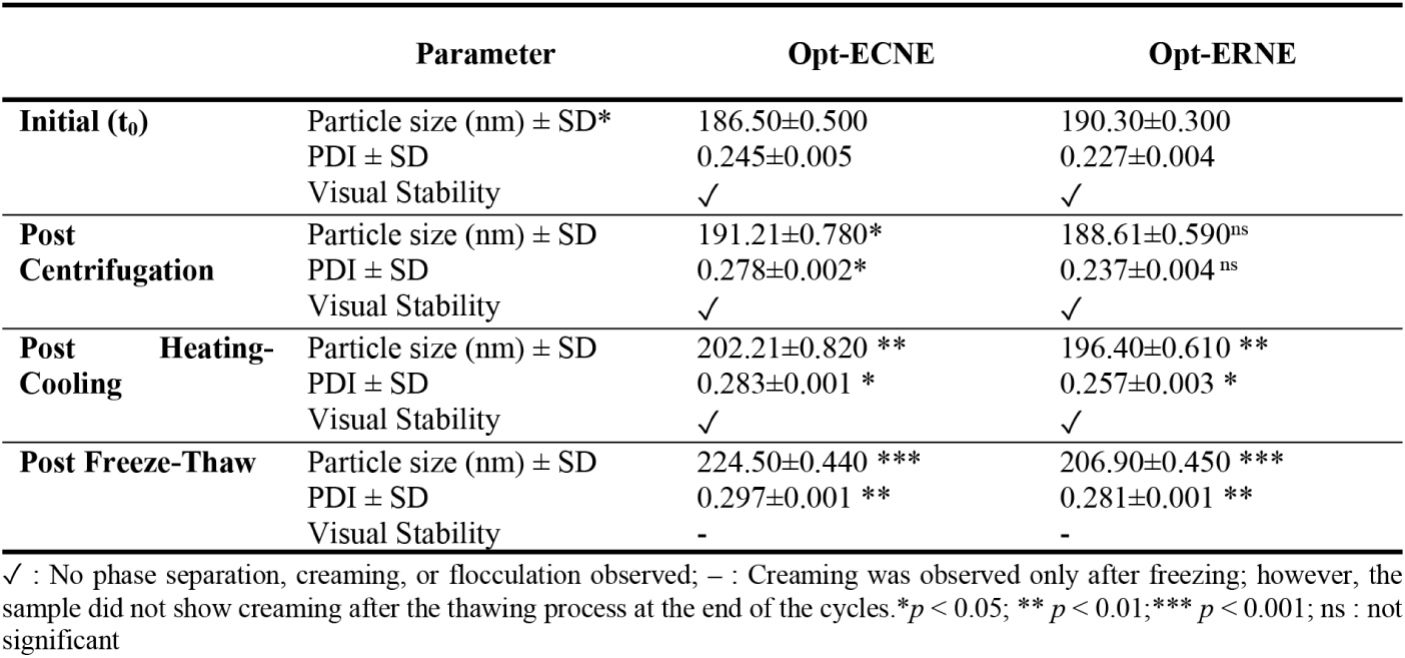
Thermodynamic Stability Assessment of Opt-ECNE and Opt-ERNE Nanoemulsions under Accelerated Conditions

DLS data verified these observations, revealing minimal fluctuations in droplet size and PDI, indicative of preserved internal structure and interfacial stability. Among the applied stress conditions, the freeze–thaw cycles imposed the most pronounced destabilizing effect, producing transient increases in droplet diameter and PDI, along with reversible creaming that disappeared upon thawing. Interestingly, Opt-ERNE exhibited slightly superior resistance to such thermal and mechanical stresses, which may be attributed to the stabilizing influence of its specific surfactant-to-oil ratio. Overall, both formulations retained droplet sizes below 200 nm and PDI values under 0.3, confirming their strong physical and colloidal stability under accelerated conditions.^24,104^ Consistent with these findings, representative photographs supporting the macroscopic stability of Opt-ECNE and Opt-ERNE under accelerated conditions are provided in Supplementary Figure S2.

### 3.9 Antibacterial Activity

MIC of *E. citriodora* EO against *S. aureus*, *E. faecalis*, and *K. pneumoniae* was 37.5, 75, and 37.5 mg/mL, respectively. On the other hand, *E. radiata* EO had an MIC of 18.75 mg/mL against *K. pneumoniae*, whereas no antibacterial activity was detected at the highest concentration (150 mg/mL) against *S. aureus* and *E. faecalis*. The EO of *E. citriodora* had a slightly higher activity against *S. aureus* and *E. faecalis*, but the activity of the oil was inferior to some extent against *K. pneumoniae* when compared to the EO of *E. radiata* (Table 8). The highest concentrations of the EOs and the nanoemulsions that were used alone as negative control showed no antibacterial activity.

**Table 8:** Minimum Inhibitory Concentrations (MICs) of the EOs and the Optimized Nanoemulsions^d^

	*E. citriodora*			*E. radiata*		
	MIC (mg/mL)				MIC (mg/mL)	
	EO^b^	Opt-ECNE^c^	Decrease in MIC (fold)^a^	EO^b^	Opt-ERNE^c^	Decrease in MIC (fold)^a^
*S. aureus*	37.5 ± 0.0	1.17 ± 0.0	32	>150 ± 0.0	18.75 ± 0.0	>8
*E. faecalis*	75 ± 0.0	2.34 ± 0.39	32	>150 ± 0.0	2.34 ± 0.39	>64
*K. pneumoniae*	37.5 ± 0.0	4.68 ± 0.78	8	18.75 ± 0.0	9.37 ± 0.0	2

a Fold-decrease in the MIC by nanoemulsion formulation.

b EO: Essential oil.

c Opt-ECNE: Optimized *E. citriodora* nanoemulsion; Opt-ERNE: Optimized *E. radiata* nanoemulsion.

d Data are represented as the standard error of the mean (±S.E.M).

When compared to other *Eucalyptus* species such as *E. globulus* and *Eucalyptus camaldulensis*, there is a limited number of studies focusing on the antibacterial properties of *E. radiata* and *E. citriodora*. Parallel to the findings of the present study, MIC of *E. radiata* oil was reported to range from 2 to 16 mg/mL against *K. pneumoniae*.^105,106^ Similarly, Mulyaningsih et al.^107^ reported a relatively higher MICs (more than 4 mg/mL) for the EOs of *E. citriodora* and *E. radiata* against *K. pneumoniae*. On the other hand, there are contradictory findings in the literature, reporting promising antibacterial activity of *E. citriodora* EO against *S. aureus*, *E. faecalis*, and *K. pneumoniae* with lower MICs.^108,109^ Consistent with the findings of our previous study, in which *E. radiata* EO did not show any antistaphylococcal and antienterococcal activity at 125 mg/mL, the EO of *E. radiata* exhibited no antibacterial activity against *S. aureus* and *E. faecalis* at the highest concentration (150 mg/mL) tested in the present study.^110^

The differences among the studies can be due to variations in the constituents and the preparation techniques of the EOs, differences in the methods used to detect antibacterial activity, and interstrain variabilities in the bacteria tested.^110^ *Eucalyptus* EOs exert antibacterial activity via various mechanisms, including leakage of intracellular components due to increased membrane permeability because of interaction of EOs with the cellular membrane, disruption of biochemical pathways via denaturation of intracytoplasmic proteins and bacterial enzymes, and ion chelation. The spectrum of antibacterial activity of the EOs of different *Eucalyptus* species changes, depending on the variations in the major constituents.^83^ In the present study, *E. citriodora* EO had slightly higher activity against *S. aureus* and *E. faecalis* compared to *E. radiata*. Similar to the findings of the present study, *E. citriodora* EO and its main component, citronellal, were reported to be more effective against antibiotic-resistant *S. aureus* strains than the oil of *E. radiata* and its main compound 1,8-cineol.^107^ On the other hand, the finding in the present study that the antibacterial activity of *E. radiata* EO is higher than that of *E. citriodora* EO against *K. pneumoniae* indicates that not only the constituents of the EO but also the intergenus differences, mainly the differences in the cell wall structure, determine the antibacterial activity.

The antibacterial activities of the Opt-ECNE against the tested bacteria were 8–32-fold higher than those of the EOs alone. MIC of Opt-ERNE was 18.75 mg/mL against *S. aureus*, 2.34 mg/mL against *E. faecalis*, and 9.37 mg/mL against *K. pneumoniae*, with an overall 2–64 times higher activity compared to the EOs (Table 8). Opt-ECNE had a slightly higher activity than Opt-ERNE against *S. aureus* and *K. pneumoniae*. Emulsions prepared without the EOs did not show any antibacterial activity, highlighting that the antibacterial activity belongs to the EOs.

Among natural products of aromatic plants, EOs of *Eucalyptus* species are well-known for their diverse biological activities, including antibacterial properties. On the other hand, high volatility, poor solubility in water, and instability limit their therapeutic applications. As a novel delivery system, nanoemulsion formulations have gained significant interest, overcoming the disadvantages and increasing the biological activity and bioavailability of EOs.^111,112^ The great majority of *Eucalyptus* EO nanoemulsions reported in the literature belonged to formulations containing the oil of *E. globulus*. Alipanah et al.^113^ reported that *E. globulus* EO nanoemulsion was effective against *S. aureus* at a concentration of 5 mg/mL. Antibacterial activity of the Opt-ECNE detected in our study against *S. aureus* (MIC of 1.17 mg/mL) was superior, whereas that of Opt-ERNE (MIC of 18.75 mg/mL) was slightly lower than that reported by Alipanah et al.^113^ In another study, *Eucalyptus* EO nanoemulsion was found to have long-lasting inhibitory action against *S. aureus* with an MIC value of 15 mg/mL, which is comparable to the antibacterial activity of Opt-ERNE but lower than that of Opt-ECNE detected in the present study. When compounded with carboxymethyl chitosan and carbomer, the nanoemulsion containing eucalyptus oil was shown to decrease the microbial load on wounds infected with *S. aureus*.^114^ Similarly, *Eucalyptus* oil nanoemulsion and chitosan-impregnated oil nanoemulsion were found to have wound healing properties and potent activity against *S. aureus*.^80^ *Eucalyptus* oil nanoemulsions prepared using the ultrasonic technique were reported to have higher antibacterial activity against *Escherichia coli* than the oil alone.^28^ Data related to *Eucalyptus* species other than *E. globulus* in the literature are limited.^109^ A nanoemulsion containing *E. citriodora* EO was effective against *Streptococcus mutans*, a cariogenic bacterium, with a slightly lower MIC than *E. globulus* EO nanoemulsion. Mouthwashes consisting of the nanoemulsion of the EO of *E. citriodora* exhibited a remarkable antibacterial effect, promising to be a complement for oral health.^83^ Although comparative studies investigating antibacterial activity of *Eucalyptus* EOs and their nanoemulsions are lacking, many of the studies investigating cinnamon, clove, thyme, turpentine, lavender, and basil oils indicated superior antibacterial activity toward nanoemulsions.^115–117^ Moreover, nanoemulsion formulations were reported to enhance the antibacterial activity of cineole, one of the main compounds of *E. radiata*, against *S. aureus*, *E. faecalis*, and *Streptococcus pyogenes*.^118^ The increased antibacterial activity of EO containing nanoemulsions was attributable not to a difference in the action of mechanism but to their small particle sizes. In parallel to the findings of the present study, droplets lower than 200 nm in size were reported to exert higher antibacterial activity. The increased surface area due to smaller particle size expands the contact of the EO with the bacterial cell membrane, triggering profound cellular defects.^112,119,120^

A slightly higher antibacterial activity of Opt-ECNE compared to Opt-ERNE against *S. aureus* and *K. pneumoniae* may be attributed to the relatively smaller droplet size of the Opt-ECNE nanoemulsion. Nevertheless, the observation that both formulations exhibited comparable antibacterial activity against *E. faecalis* suggests that the efficacy of these systems is not solely governed by particle size. Other contributing factors such as the specific composition of the EOs and the intrinsic characteristics of the bacterial genus also play a crucial role in determining the overall antibacterial performance.

### 3.10 Biofilm Inhibition

*E. radiata* EO showed a promising antibiofilm activity against *S. aureus*, inhibiting the biofilm formation by around 77% at 37.5 (1/8 × MIC) and 75 mg/mL (1/4 × MIC), respectively. On the other hand, the EO of *E. citriodora* did not have any significant activity against the biofilm formation by *S. aureus*. The biofilm inhibition by the EO of *E. radiata* against *E. faecalis* was 58% at 1/2 × MIC (150 mg/mL). Likewise, *E. citriodora* EO inhibited *E. faecalis* biofilm formation at 37.5 mg/mL (1/2 × MIC) by 62%. The EO of both *E. citriodora* and *E. radiata* did not show any significant biofilm inhibition activity against *K. pneumoniae* (Figures 10–12).

**Figure 10:**
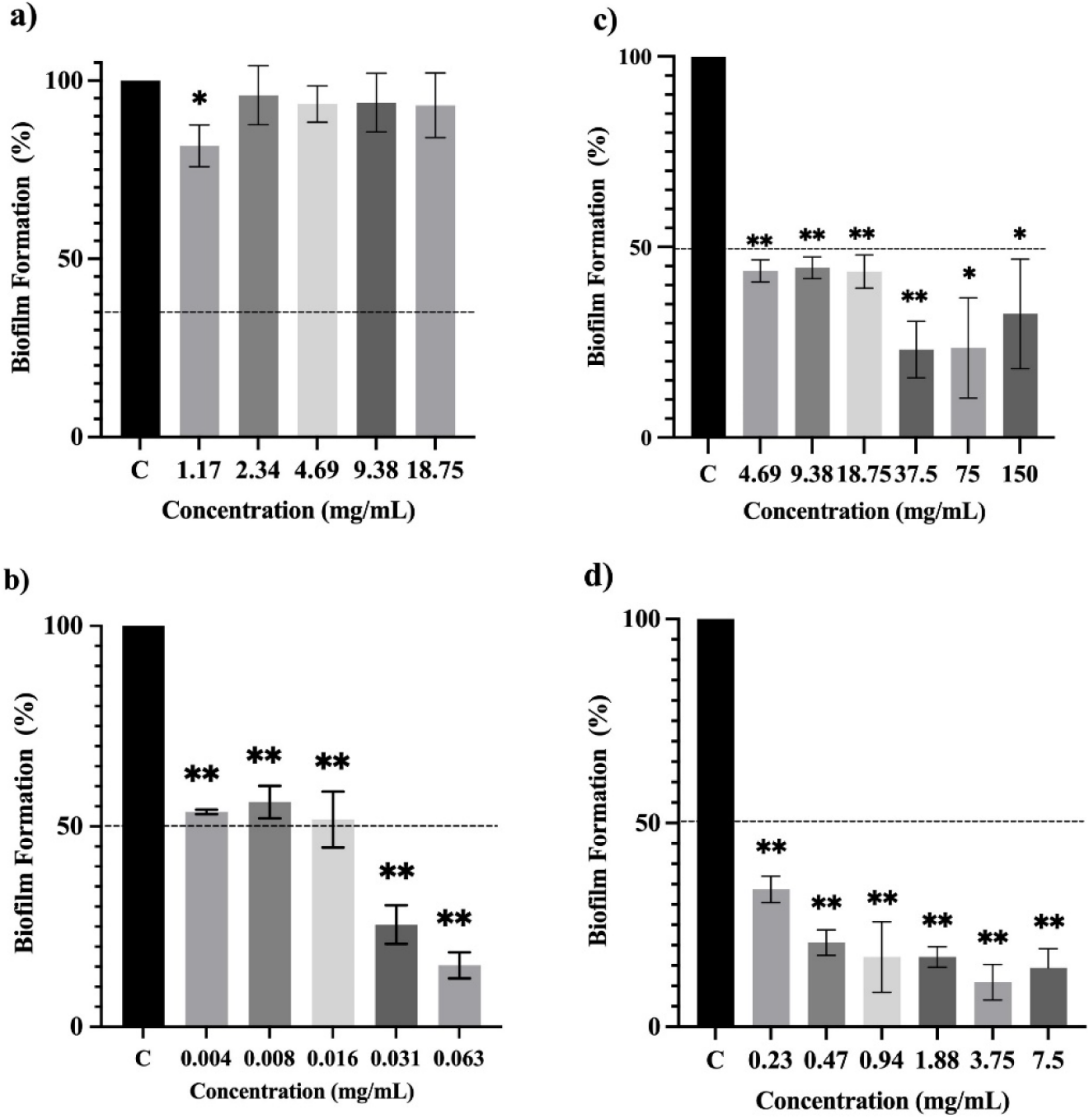
Antibiofilm activity of the essential oils and nanoemulsion formulations against *Staphylococcus aureus* in comparison to growth controls (C). (a) *E. citridiora* essential oil, (b) *E. citriodora* *essential oil* nanoemulsion, (c) *E. radiata* essential oil, (d) *E. radiata* essential oil nanoemulsion. Data represented as mean ± SEM (*n* = 3; * = *p* < 0.05 and ** = *p* < 0.01).

Formation of a biofilm, a niche where one or more than one species of microorganisms is enclosed by an extracellular matrix and consisting of polysaccharides, proteins, nucleic acids, and water, is a population-level virulence factor adapted by bacteria, preventing them from the effect of harsh, physical, chemical, and biological environmental factors such as ultraviolet irradiation, antibiotics, disinfectants, and the attack of immune cells. In addition to food and pharmaceutical industries, biofilms, thought to be responsible for 80% of bacterial infections, pose a health threat in clinical settings for the treatment of bacterial infections.^121–123^ EOs are promising natural products for the inhibition of biofilm formation and the eradication of preformed biofilms, preventing biofilm-related infections or improving the antibiotic treatment of biofilm-associated infections.^123^ Various studies investigated the antibiofilm activity of *Eucalyptus* spp. against *S. aureus*, a bacterium that is frequently isolated from wound and catheter-related infections. *E. globulus* EO inhibited the biofilm formation by methicillin-resistant *S. aureus* (MRSA) at a concentration of 1/16 x MIC.^124^ In another study, *E. globulus* revealed a 50% reduction in biofilm formation against *S. aureus* at 25 mg/mL.^125^ All seven different *Eucalyptus* species other than *E. globulus*, *E. radiata*, and *E. citriodora* were reported to have antistaphylococcal biofilm activity ranging from 59 to 95% at 20 μL/mL.^126^ Although EO of *E. radiata* had a slightly lower antibacterial activity against *S. aureus* than that of *E. citriodora*, antibiofilm activity of *E. radiata* EO was superior, indicating differences in the mechanism of action between antibacterial activity and biofilm inhibition. The biofilm inhibition activity of *E. radiata* detected in the present study against *S. aureus* and *E. faecalis* at sub-MIC concentration is promising for the prevention of catheter-associated infections and wound infections in clinical settings, and food spoilage in food industry, warranting further investigation.

*Eucalyptus* oil was reported as a promising adjunctive strategy to normal preventive measures for biofilm-related caries and endodontic infections due to its significant reduction in the biofilm formation by *S. mutans* and *E. faecalis*.^127,128^ In the present study, the finding that the biofilm formation by *E. faecalis*, a bacterium which can be responsible for endodontic and nondevice and device-related biofilm infections such as wound, catheter-related urinary tract and bloodstream infections, was inhibited by the EOs at sub-MIC concentrations is promising for fighting against the biofilm-related infections. As far as we investigated, no study compared the antibiofilm activity of *E. radiata* and *E. citriodora* EOs at their subinhibitory concentrations against *S. aureus*, *E. faecalis*, and *K. pneumoniae*, highlighting the importance of the present study.

Major compounds of aromatic plants, such as terpenes including eucalyptol, linalool, and limonene, and phenolic components like carvacrol, thymol, and eugenol, were shown to have antibiofilm activity. Although the action of EOs as biofilm inhibitors includes various mechanisms such as inhibition of exopolysaccharide matrix formation, attachment, and metabolic activity, the main mechanism of action, especially at subinhibitory concentrations, is attributed to the inhibition of quorum-sensing, a way of communication among bacteria depending on the cell density via chemicals called autoinducers. A limitation of the present study is that the mechanistic evidence underlying the antibacterial and antibiofilm activities has not been investigated. However, previous studies have shown that the majority of natural products exert their antibacterial effects by disrupting the bacterial cell membrane. This disruption reduces the viability of adherent cells and inhibits bacterial adhesion, ultimately impairing biofilm formation.^129,130^ Furthermore, quorum sensing is important for the regulation of biofilm formation.^121,131^ Antiquorum sensing activities of *E. radiata* and *E. globulus* EOs were reported in various studies.^124,132,133^ *E. globulus* was one of 15 EOs that was reported to be a promising alternate intervention strategy against multidrug-resistant*K. pneumoniae* infections due to its antiquorum sensing activity.^128^

Opt-ECNE led to a decrease in the biofilm formation by *S. aureus* from 46% to 84% at lower sub-MIC concentrations ranging from 0.004 to 0.063 mg/mL (1/2 × MIC and 1/32 × MIC, respectively) when compared to the EO. Similarly, the biofilm inhibition rate of Opt-ERNE (ranging from 66 to 89%) against *S. aureus* was higher than that of the *E. radiata* EO at much lower concentrations (0.23–3.75 mg/mL) (Figure 10). Antibiofilm activity of the Opt-ECNE against *E. faecalis* ranged at high rates from 64 to 88% in a dose-dependent manner, which was superior in comparison to the oil alone at lower sub-MIC concentrations. ERNE increased the maximum enterococcal biofilm inhibition by the EO from 62% at 1/2 × MIC (37.5 mg/mL) to around 50% at lower concentrations, which are 4- to 32-fold less than the MIC of the formulation (Figure 11). Like their EOs, ECNE and ERNE did not show any significant antibiofilm inhibition activity against *K. pneumoniae* (Figure 12).

**Figure 11:**
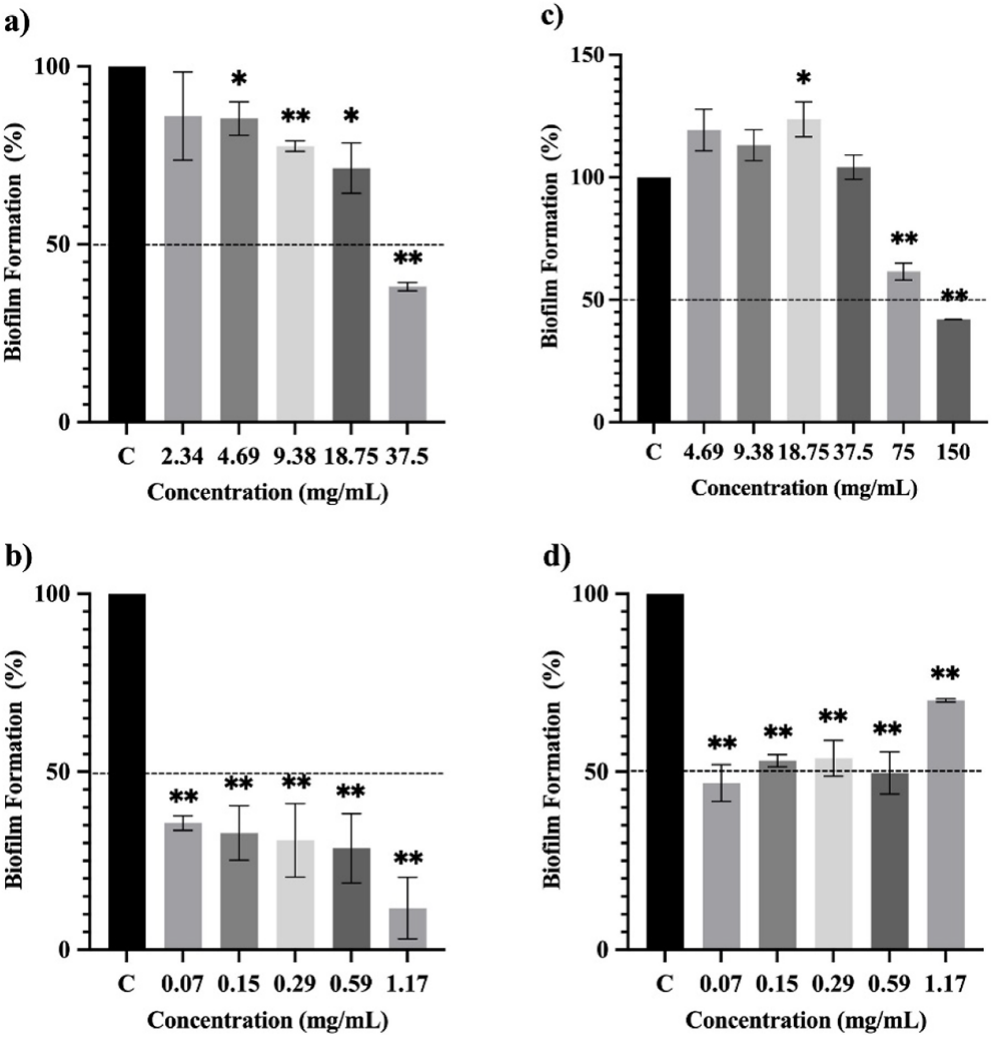
Antibiofilm activity of the essential oils and nanoemulsion formulations against *Enterococcus faecalis* in comparison to growth controls (C). (a) *E. citriodora* essential oil, (b) *E. citriodora* essential oil nanoemulsion, (c) *E. radiata* essential oil (d) *E. radiata* essential oil nanoemulsion. Data represented as mean ± SEM (*n* = 3; * = *p* < 0.05 and ** = *p* < 0.01).

**Figure 12:**
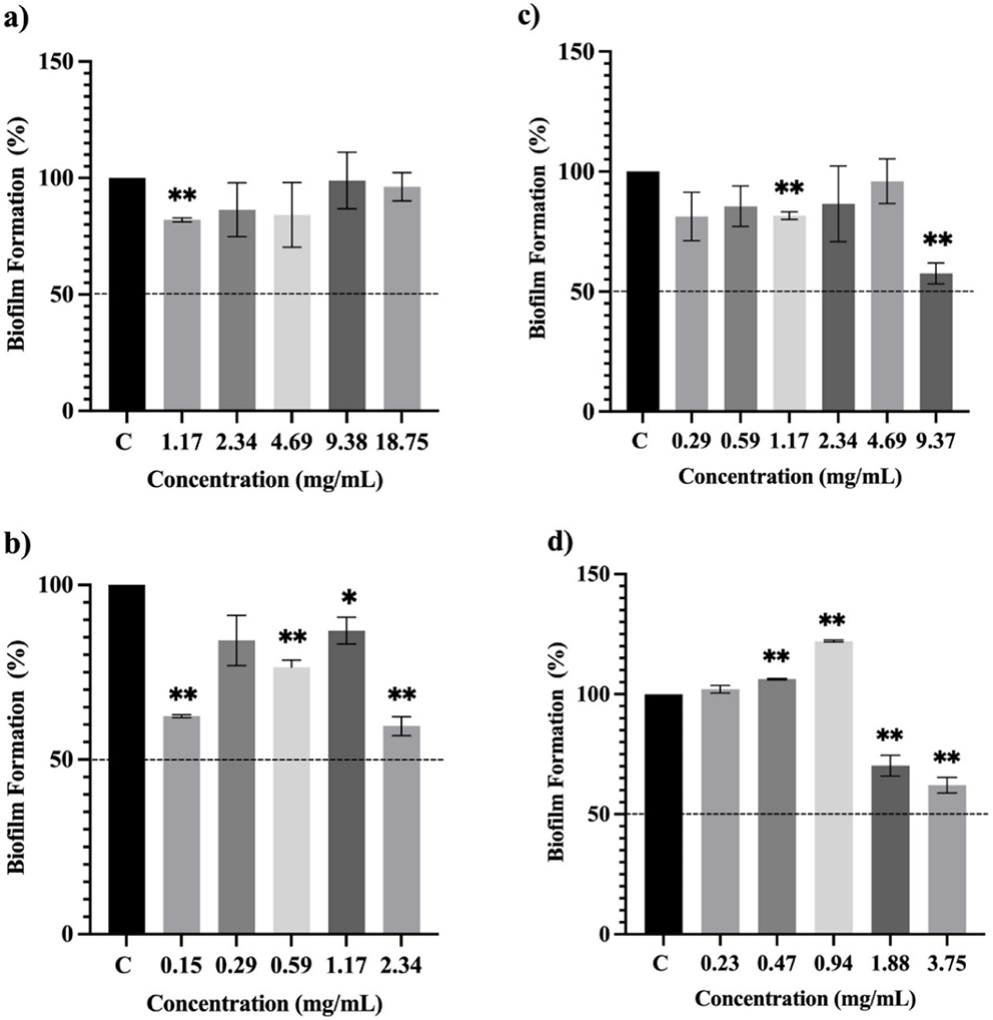
Antibiofilm activity of the essential oils and nanoemulsion formulations against *Klebsiella pneumoniae* in comparison to growth controls (C). (a) *E. citriodora* essential oil, (b) *E. citriodora* essential oil nanoemulsion, (c) *E. radiata* essential oil, (d) *E. radiata* essential oil nanoemulsion. Data represented as mean ± SEM (*n* = 3; * = *p* < 0.05 and ** = *p* < 0.01).

Nanoemulsions are a novel method for delivering EOs to combat biofilm formation, as they are both thermodynamically and kinetically stable.^134^ Consistent with the findings of our study, Cai et al.^114^ reported that a nanoemulsion containing *E. globulus* EO inhibited *S. aureus* biofilm formation by 77% at 30 mg/mL (2 × MIC) and by around 60% at 7.5 mg/mL (1/2 × MIC). Clove oil nanoemulsion exhibited approximately 4–5 times higher antibiofilm activity against *S. aureus* at 1/2 × MIC and 1/4 × MIC concentrations compared to the emulsion formulation.^116^ Similarly, nanoemulsions containing the EO of *Origanum vulgare* revealed a superior biofilm inhibition activity at concentrations two to four times lower than the EO alone against foodborne pathogens, including *S. aureus*, *E. coli*, *L. monocytogenes*, and *S. enteritidis*.^135^ In parallel to the findings of the present study that highlights a higher antibiofilm activity against *S. aureus* and *E. faecalis* by nanoemulsions compared to the EOs, Hashem et al.^136^ reported a superior antibiofilm activity against *S. aureus* by nanoemulsion formulations including thyme and clove EO than the EOs alone. An increase in biofilm inhibition by nano formulations at subinhibitory concentrations can be explained by a rise in interaction between the EOs and bacteria due to the greater surface area of the particles which decreases the biofilm formation capacity of microorganisms via the inhibition of quorum sensing that regulates the expression of various bacterial structures such as flagella, pili, and exopolysaccharides.

## 4 Conclusion

This study systematically compared and optimized nanoemulsion formulations incorporating three *Eucalyptus* EO, elucidating how compositional diversity governs colloidal dynamics and antimicrobial efficacy. The BBD enabled precise tuning of formulation variables, resulting in nanosystems with sub-200 nm droplets, narrow PDI (<0.3), and superior physical stability. Nanoencapsulation substantially enhanced antimicrobial potency, achieving MIC values as low as 0.625 mg/mL and up to 80% inhibition of biofilm formation at sub-MIC levels against *S. aureus* and *E. faecalis*. By overcoming the intrinsic volatility and instability of EOs, the developed nanoemulsions demonstrate a robust, scalable, and sustainable delivery platform. Overall, these findings highlight the translational promise of Eucalyptus-based nanocarriers as next-generation antimicrobial systems capable of addressing persistent and biofilm-associated infections. To advance these optimized nanoemulsions toward more patient friendly use, future work will focus on incorporating them into more wound-appropriate topical semisolid dosage forms (e.g., nanoemulgels) and conducting cell-based biocompatibility assessments, with subsequent *in vivo* evaluation as appropriate, to support translational application.

## Supplementary Material



## Notes

Figure S1 (long-term stability): representative photographs of Opt-ECNE and Opt-ERNE documenting macroscopic appearance on days 1, 30, and 90. Figure S2 (accelerated stability): representative photographs of Opt-ECNE and Opt-ERNE documenting macroscopic appearance on days 1, 30, and 90 (PDF)
