# Supplementary material for: Nanoencapsulation of Eucalyptus Essential Oils via Box–Behnken Design: Phytochemical Profiling and Enhanced Antibacterial and Antibiofilm Efficacy
Source: ACS Omega. 2026 Feb 16;11(8):13799–818. doi: 10.1021/acsomega.5c11998 (PMC12961624; doi:10.1021/acsomega.5c11998)
Supplement: Supplementary file 1 [file ao5c11998_si_001.pdf]

Supporting Information

Nanoencapsulation of *Eucalyptus* Essential Oils via Box–Behnken Design: Phytochemical Profiling and Enhanced Antibacterial and Antibiofilm Efficacy

Leyla Beba Pozharani[a]*, Mehmet İlktaç[a], Ezgi Ak-Sakallı[b], Mustafa Alhadi[a], Ertugrul Ozbil[a], Azmi Hanoglu[c], Fatih Demirci[d], Murat Erdem[e], Kemal Husnu Can Baser[c] and Muberra Kosar[a]

[a] Eastern Mediterranean University, Faculty of Pharmacy, 99628 Famagusta, North Cyprus, via Mersin 10, Turkiye
[b] Final International University, Faculty of Pharmacy, 99320 Kyrenia, North Cyprus, via Mersin 10, Turkiye
[c] Near East University, Faculty of Pharmacy, Department of Pharmacognosy, 99138 Nicosia, North Cyprus, via Mersin 10, Turkiye
[d] Anadolu University, Faculty of Pharmacy, Department of Pharmacognosy, 26470 Eskisehir, Turkiye
[e] Eskisehir Technical University, Faculty of Science, Department of Chemistry, Yunusemre Campus, 26470 Eskisehir, Turkiye

***Corresponding Author**
Leyla Beba Pozharani
leyla.beba@emu.edu.tr
ORCID iD: 0000-0001-5051-4489

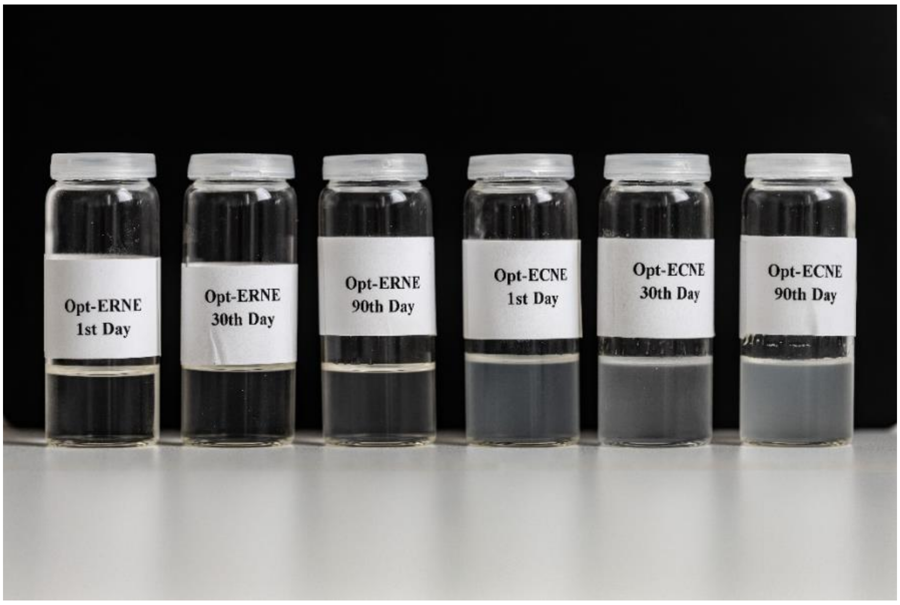

Figure S1. Stability test image of Opt-ECNE and Opt-ERNE formulations on days 1, 30, and 90 of storage.

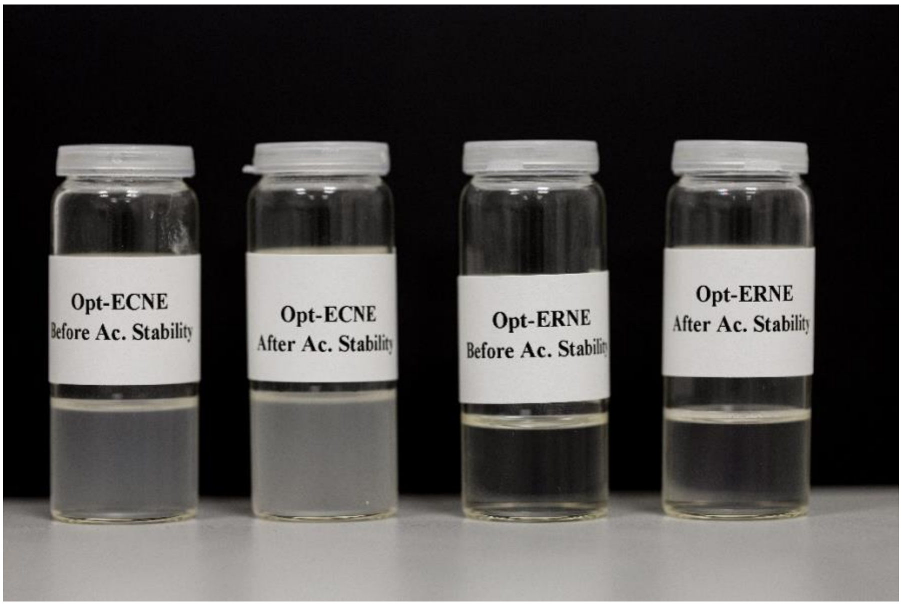

Figure S2 Image of Opt-ECNE and Opt-ERNE formulations under accelerated storage conditions.
